# Scelestial: Fast and accurate single-cell lineage tree inference based on a Steiner tree approximation algorithm

**DOI:** 10.1371/journal.pcbi.1009100

**Published:** 2022-08-11

**Authors:** Mohammad-Hadi Foroughmand-Araabi, Sama Goliaei, Alice C. McHardy

**Affiliations:** 1 Computational Biology of Infection Research, Helmholtz Centre for Infection Research, Braunschweig, Germany; 2 Braunschweig Integrated Centre of Systems Biology (BRICS), Technische Universität Braunschweig, Braunschweig, Germany; University of Michigan, UNITED STATES

## Abstract

Single-cell genome sequencing provides a highly granular view of biological systems but is affected by high error rates, allelic amplification bias, and uneven genome coverage. This creates a need for data-specific computational methods, for purposes such as for cell lineage tree inference. The objective of cell lineage tree reconstruction is to infer the evolutionary process that generated a set of observed cell genomes. Lineage trees may enable a better understanding of tumor formation and growth, as well as of organ development for healthy body cells. We describe a method, Scelestial, for lineage tree reconstruction from single-cell data, which is based on an approximation algorithm for the Steiner tree problem and is a generalization of the neighbor-joining method. We adapt the algorithm to efficiently select a limited subset of potential sequences as internal nodes, in the presence of missing values, and to minimize cost by lineage tree-based missing value imputation. In a comparison against seven state-of-the-art single-cell lineage tree reconstruction algorithms—BitPhylogeny, OncoNEM, SCITE, SiFit, SASC, SCIPhI, and SiCloneFit—on simulated and real single-cell tumor samples, Scelestial performed best at reconstructing trees in terms of accuracy and run time. Scelestial has been implemented in C++. It is also available as an R package named RScelestial.

## Author summary

Reconstructing the evolutionary history from the genome sequences of single cells can provide a detailed understanding of evolutionary events and changes on a very fine-grained scale, for instance in the development of organs and cancer. Due to the increasing sizes of single-cell datasets, scalable and accurate methods are required. In this work we describe Scelestial, a software implementing an adapted Steiner tree approximation algorithm for evolutionary tree reconstruction from the analysis of single-cell datasets. The Steiner tree approximation algorithm, unlike other heuristics and sampling-based methods (e. g. Markov chain Monte Carlo), provides guarantees of its performance. A comparison of Scelestial with state of the art methods showed that it performed favourably in terms of quality of the inferred trees as well as speed across a large number of simulated data sets, and produced the most plausible evolutionary scenarios on single cell data sets from cancer patients. Taken together, our results show that Scelestial provides a valuable addition to current single cell lineage inference techniques.

This is a *PLOS Computational Biology* Methods paper.

## 1 Introduction

Lineage trees describe the evolutionary process that created a sample of clonally related entities, such as individual cells within an organ or a tumor. A lineage tree also suggests the constitution of a founder cell, as well as of its descendants, and the evolutionary events that occurred during lineage formation. Single-cell genomic data provide a highly resolved view of cellular evolution, substantially more than bulk genome sequencing [1]. However, they also come with high rates of missing values and distorted allele frequencies arising from amplification biases and sequencing errors [2]. Multiple displacement amplification (MDA) is a commonly used single-cell DNA sequencing that is suitable for single-nucleotide polymorphism identification. Lineage tree reconstruction from single-cell data therefore requires specific considerations for handling missing values and errors. Specific approaches to tackle this problem include the methods of Kim and Simon [3], BitPhylogeny [4], OncoNEM [5], SCITE [6], SiFit [7], SASC [8], SPhyR [9], SCIPhI [10], SiCloneFit [11], B-SCITE [12], and PhISCS [13]. Kim and Simon [3] make use of the infinite site assumption and infer a “mutation tree” based on calculating a probability for ordering mutations in a lineage and constructing a tree, finding the maximum spanning tree in this graph. BitPhylogeny [4] provides a stochastic process through a graphical model that stochastically generates the given input data and uses Markov Chain Monte Carlo (MCMC) for sampling to search for the best lineage tree model and the associated parameters. OncoNEM [5] and SCITE [6] infer a phylogenetic tree over all the samples under a maximum likelihood model. OncoNEM uses a heuristic search, whereas SCITE uses MCMC sampling to find a maximum likelihood tree. SiCloneFit [11] infers subclonal structures and a phylogeny via a Bayesian method under the finite site assumption. SASC [8] and SPhyR [9] consider the k-Dollo model, a more relaxed model in comparison than infinite site assumption. In k-Dollo model, a mutation can be gained once in a tumor but may be lost multiple times afterwards. SASC uses simulated annealing and SPhyR uses k-means to find the best k-Dollo evolutionary tree. B-SCITE [12] and PhISCS [13] infer subclonal evolution, based on a combination of single-cell and bulk sequencing data. B-SCITE uses an MCMC method to maximize a likelihood function for trees and sequencing data. PhISCS formulates tree reconstruction as combinatorial and mathematical programming problems, and uses standard mathematical programming solvers to find the solution. Integer or integer linear programmings for the phylogenetic tree reconstruction are also available [14, 15].

The Steiner tree problem is a classic problem in theoretical computer science with a wide range of applications in various areas including very-large-scale integration design [16], network routing [17], civil engineering [18], and other areas [19–22]. Given a weighted graph and some vertices marked as terminals, the Steiner tree problem is the problem of finding the minimum weighted tree that connects all the terminal vertices, with non-terminal vertices that may or may not be included in the optimal tree. Although the Steiner tree problem is NP-hard and no polynomial-time exact solution is known, some elegant approximation algorithms are available. They guarantee polynomial time and a constant factor approximation ratio, and have been used for phylogenetic reconstruction [23]. This is a great advantage compared with sampling heuristics such as MCMC for finding an optimal solution, which may be trapped in local optima.

Here, we describe Scelestial, a method for lineage tree reconstruction from single-cell datasets, based on the Berman approximation algorithm for the Steiner tree problem [24]. Our method infers the evolutionary history for single-cell data in the form of a lineage tree. Dealing with missing values makes the problem much harder in theory. To overcome this difficulty, we considered a geometrical representation of the problem. Considering two-state sequences, the sequences can be represented as data points in a high dimensional space such that dimensions correspond to sequence loci. In this representation the phylogeny inference problem could be considered as a geometric Steiner tree problem, in which weight of edges are calculated as the Euclidean distances between the points. The idea of the definition of the cost function in Scelestial regarding the missing values is to put a new point for each sequence at the center of mass of the points representing all the potential imputations of a sequence. Considering this geometrical representation, although the definition of cost function imposes some extra cost in the result of the algorithm caused by misplacement of the center from the best imputation, which we do not know in advance, helps us to get a fast yet accurate and robust algorithm.

## 2 Results

Scelestial is a phylogenetic inference algorithm based on an approximation algorithm for the Steiner tree problem that has been adapted for single-cell data. Scelestial’s input is a set of single-cell genome sequences given as a matrix of point mutations, which may contain missing values. Scelestial defines a cost function for every pair of sequences and starts with a minimum cost spanning tree between the samples, and considers it as a first-order approximation of the inferred phylogeny. Scelestial then iteratively improves the inferred tree by considering all subsets of samples of a size up to a constant parameter *k* and all the potential phylogenies containing these samples as their leaf nodes. The output is an unrooted phylogenetic tree representing the evolutionary relationships between sampled cells.

**Fig 1:**
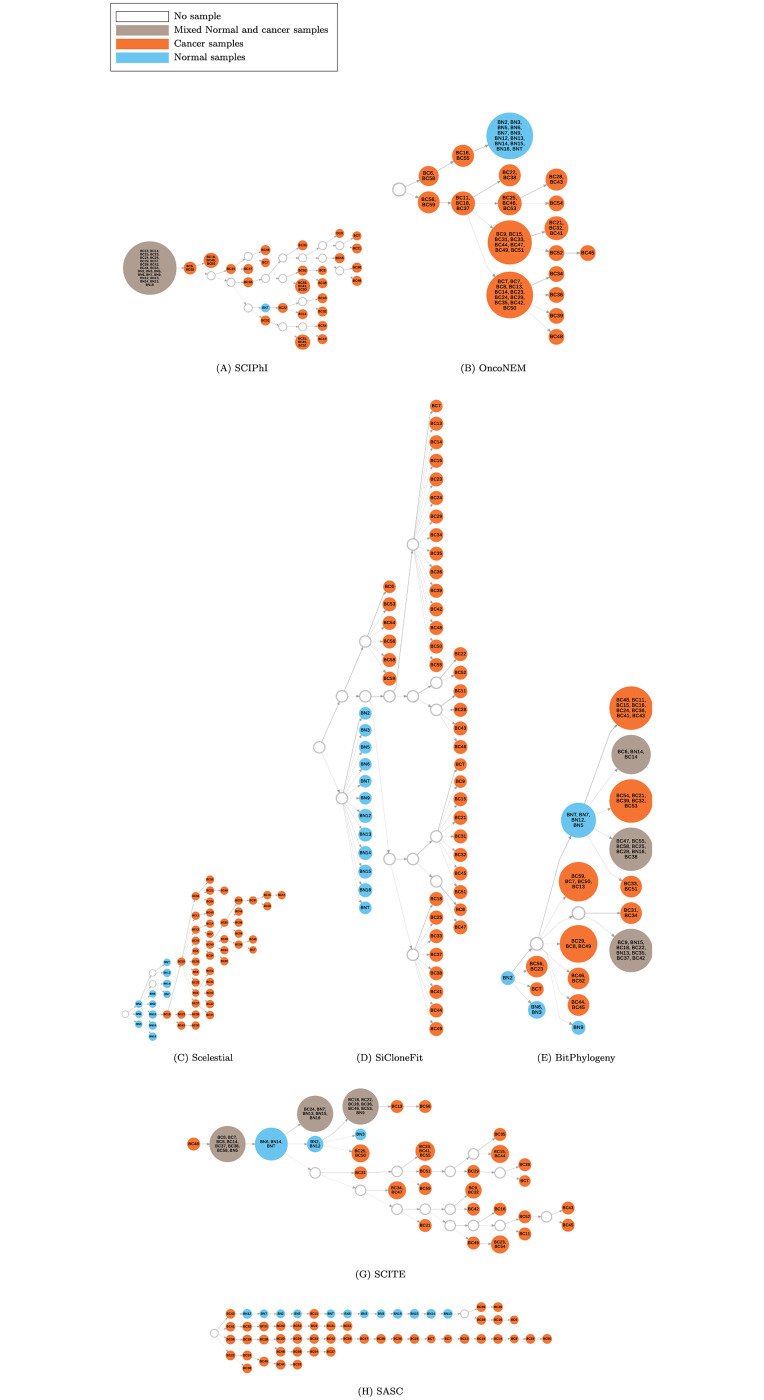
Lineage trees inferred by different methods on a single-cell dataset from a muscle-invasive bladder tumor. Nodes in these trees represent clones (i.e., inferred, evolutionary genomes ancestral to the observed single-cell genomes). For the methods generating trees over samples, and not the clones, colored nodes represent samples. Blue nodes represent clones containing only normal cells, orange nodes represent clones containing only cancer cells, and brown nodes represent clones containing both normal and cancer cells. Text within the nodes indicates the identification number of the assigned sample cell(s) to the corresponding clone. White nodes represent nodes with no observed sample.

Phylogenetic inference methods produce trees with samples assigned only to leaf nodes. However, in single cell analysis some methods (e.g. SASC [8]) produce a tree allowing samples to be assigned to internal nodes. An assignment of a sample to an internal node indicates an ancestral node with a state indistinguishably similar to that sample (Fig 1A, 1B, 1E, 1G and 1H). It could equally well be represented by a tree with samples assigned only to terminal nodes by adding a branch with zero edge length leading to that sample to this node. Scelestial follows the single-cell convention.

Since Scelestial reconstructs a phylogeny by considering the similarity of the sample nucleotides in variable positions and therefore does not need to be given information on the reference alleles as input. However, Scelestial does make use of this information when using the option for specifying the root of the phylogeny, which is then identified as the sample most similar to the reference (normal) cells.

### 2.1 Performance in lineage tree reconstruction on simulated data

We compared the performance of Scelestial to SCITE, OncoNEM, BitPhylogeny, SASC (as a recent instance of k-Dollo-based methods), SCIPhI, SiFit, and SiCloneFit. For this, we generated data with the cell evolution simulator provided by OncoNEM and with another tumor evolution simulator that we developed (Section 3.2).

The methods evaluated in this study were developed under different assumptions and evolutionary models. However, in a more general view, the sampled cells are formed through an evolutionary process. Thus, there is an actual evolutionary tree relating all of them in the real world, and the ultimate goal of all of the methods and models is to infer the true evolutionary relation between the samples. Thus, we evaluated the performance of the algorithms via comparing their resulting tree with the ground truth, on the simulated data. For the clone-based trees, we considered each clone as an internal node with all its samples as its direct descendants with zero length edges. With this consideration, the measures used for evaluation of the trees could be used for both clone-based and sample-based models.

We calculated their distance to the ground truth lineage trees as the normalized pairwise distances between corresponding samples, as described in Section 3.4.1. In addition, we calculated the similarity between the generated trees and the ground truth by comparing tree splits (Section 3.4.2). For run time evaluation, we executed all the methods on simulated tumor data over a range of numbers of samples and sites. We also assessed the run time performance of the methods in relation to different parameters, such as the number of samples and sites (Section 2.6).

First, we evaluated the algorithms on data created with OncoNEM’s simulator. The simulator creates samples by producing clones and sampling from these clones (Section 3.3). It accepts the number of clones (i.e., the number of nodes in the lineage tree), the number of sampled cells, the number of sites, the false positive rate, the false negative rate, and the missing value rate as parameters. We used 1.5% for false positive, 10% for false negative and 7% for the missing value rates. These parameters are consistent with observed parameters in single-cell data [25]. We performed tests for 50 and 100 samples, 5 and 10 clones, and 20 and 50 sites, and calculated the pairwise distance between the inferred and ground truth trees for all methods (Table 1). For each setting we evaluated the results for 10 different simulations and calculated the average (Table 1). From these data, Scelestial reconstructed lineage trees with the lowest distance to the ground truth tree in 7 out of 8 cases. SiFit performed best in the remaining case.

**Table 1:** Comparison of reconstruction of ground truth lineage tree from data simulated by OncoNEM, showing the distance between the inferred trees and the ground truth for all methods across eight lineage trees. Each cell represents average of tree distance measure for ten different simulated datasets. The best results among all the methods for each evolutionary tree are shown in bold. The variance of the values are represented in brackets.

	50 samples				100 samples			
	5 clones		10 clones		5 clones		10 clones	
Method	20 sites	50 sites	20 sites	50 sites	20 sites	50 sites	20 sites	50 sites
OncoNEM	0.93 (0.03)	0.97 (0.03)	0.80 (0.01)	0.76 (0.01)	0.96 (0.02)	0.96 (0.02)	0.84 (0.01)	0.78 (0.00)
Scelestial	**0.84 (0.02)**	**0.86 (0.03)**	**0.73 (0.01)**	**0.68 (0.00)**	0.89 (0.02)	**0.88 (0.02)**	**0.76 (0.01)**	**0.71 (0.00)**
BitPhylogeny	0.96 (0.03)	0.95 (0.02)	0.94 (0.01)	1.03 (0.05)	0.97 (0.04)	1.05 (0.02)	0.94 (0.01)	0.97 (0.02)
SCITE	1.00 (0.02)	0.97 (0.02)	0.93 (0.03)	0.89 (0.03)	0.99 (0.02)	0.96 (0.02)	0.91 (0.01)	0.98 (0.05)
SASC	0.88 (0.01)	0.91 (0.02)	0.80 (0.00)	0.78 (0.00)	0.93 (0.02)	0.92 (0.01)	0.82 (0.00)	0.80 (0.00)
SCIPhI	0.94 (0.03)	1.00 (0.01)	0.92 (0.03)	0.90 (0.01)	0.96 (0.02)	1.01 (0.03)	0.96 (0.02)	0.90 (0.04)
SiFit	0.88 (0.03)	0.90 (0.02)	0.87 (0.05)	0.82 (0.09)	**0.86 (0.02)**	0.91 (0.02)	0.84 (0.03)	0.79 (0.02)
SiCloneFit	0.96 (0.03)	1.01 (0.02)	0.83 (0.01)	0.79 (0.01)	0.99 (0.02)	1.05 (0.02)	0.90 (0.02)	0.81 (0.00)

**Fig 2:**
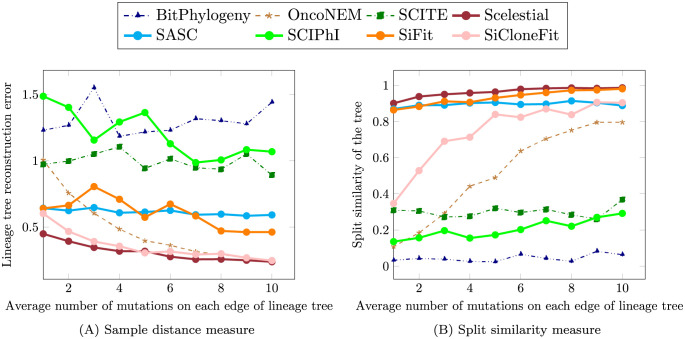
Comparison of the methods for single-cell lineage tree reconstruction on simulated tumor data. Note that in case of the lineage tree reconstruction error (A), lower values show a better reconstruction. On the other hand, the split similarity measure represents (B) similarity between the reconstructed tree and the ground truth tree, making higher values favorable.

Next, we simulated 100 single-cell datasets from a solid tumor covering a range of evolutionary time spans (i.e., a range of mutations per branch in the resulting lineage trees from one to ten), using a simulation method we implemented (Section 3.2) and compared the methods on this simulated dataset (Fig 2). The simulated data provide a granular simulation of tumor evolution and single-cell sequence data. Tumor growth was simulated with 50 samples and 200 sites. The other parameters (missing value rate, false positive rate, and false negative rate) were set as in the OncoNEM simulation. Specifically, we used 1.5% for the false positive rate, 10% for the false negative rate, and 7% for the missing value rate. The main difference between our simulation and the OncoNEM simulation tool was that we considered the relative fitness between clones and let the clones proliferate with a probability proportional to their relative fitness.

The lineage tree reconstruction error was calculated as the sum of all distance errors over every pair of samples (Section 3.4.1). The lower the lineage tree reconstruction error, the more similar the tree is to the ground truth one. In addition, we determined the split similarity between the resulting trees and the ground truth tree (Section 3.4.2). A higher split similarity measure reflects a better reconstruction of the tree’s topology. Overall, when considering the lineage tree reconstruction error, none of the resulting trees was highly similar to the ground truth tree (Fig 2A). Nevertheless, there were differences in the accuracy of the inferred trees. With these data, considering the average performance measure of 10 datasets for each mutation rate, Scelestial performed best in 10 out of 10 cases regarding the split similarity measure. Scelestial also performed best for the distance measure in 9 of 10 cases; SiCloneFit was the best for the remaining case. The performance of Scelestial, SiCloneFit, and OncoNEM improved with the number of mutations (Fig 2A). This increase in the mutation rate had a very large effect on the performance of SiCloneFit and OncoNEM, whereas the performance of SASC and SiFit was stable across changes in the number of mutations between clones.

Overall, the relative performance of lineage tree reconstruction for different methods was similar to that observed before (Table 1). This also indicates that the relative difficulty of tree inference for the simulation method provided by OncoNEM and our simulation method are similar.

### 2.2 Robustness across varying data properties

We next evaluated the robustness of Scelestial to dataset properties over 420 datasets with varying parameters that we generated for this purpose with our tumor simulator. We fixed the default configuration in our simulation as 50 samples, 200 sites, five evolutionary nodes in the simulated evolutionary tree, 1.5% as the false positive rate, 10% as the false negative rate, and 7% as the missing value rate. We set the average number of mutations between nodes in the evolutionary tree to 20. For evaluating the robustness of Scelestial with respect to one parameter, we set the other parameters to their default values and evaluated Scelestial over a range of the parameter under study. We used the following ranges: false positive rate: 0–50%; false negative rate: 0–30%; missing value rate: 0–50%; samples: 5–200; sites: 20–1000.

**Fig 3:**
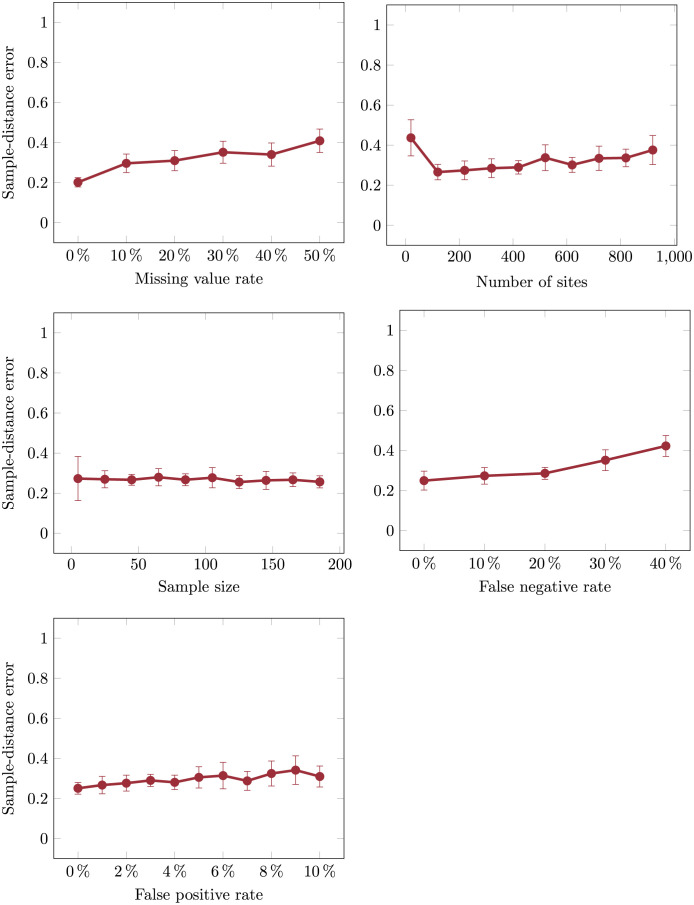
Robustness of Scelestial to variation in the properties of ground truth lineage trees in terms of sample distance in the trees between the inferred and ground truth trees.

**Fig 4:**
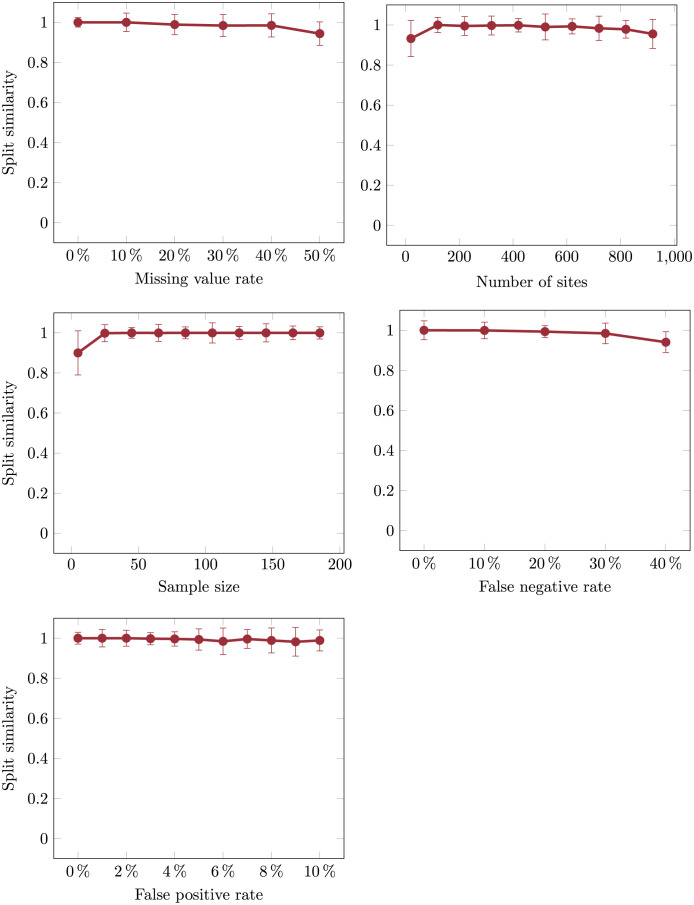
Robustness of Scelestial to variation in the properties of ground truth lineage trees in terms of topological similarity between the inferred and ground truth trees.

Scelestial was not very sensitive to variation in the tested parameters (Figs 3 and 4) in terms of their influence on the tree distance error and topological accuracy measured by split similarity. As expected, performance decreased with increasing missing value, false negative, and false positive rates (Fig 3). For datasets including more than 25 sites, the sample distance measure was stable. For all parameters except the false negative rate we observed a non-monotonic relationship to the sample-distance-error, which due to the underlying complexity of simulations was difficult to associate with any specific property or parameter.

### 2.3 Case study: A single-cell dataset from a muscle-invasive bladder tumor

We inferred lineage trees for single-cell data of a muscle-invasive bladder tumor [26] with OncoNEM, BitPhylogeny, SCITE, SASC, SCIPhI, SiFit, and SiCloneFit. The data was captured via whole-genome amplification and then second-generation sequencing of the whole single-cell genomes. Then, samples with less than 70% coverage of the exome targets or high rate of false heterozygous variant calls on the X chromosome were removed. The resulting single-cell DNA sequence data consists of 44 tumor cells as well as 13 normal cells, and included 443 variable sites directly taken from [26]. We converted this to the required input matrix for all the methods, in which only the reference state, variant state, and missing values were specified. In the resulting matrix, 27% of the elements represented the reference state, 17% represented variant states, and 55% represented missing values. Unlike the original study [5], which includes only one normal cell, we used all cancer cells as well as 13 normal cells for lineage tree reconstruction, to see whether a distinct cancer cell lineage would become apparent in the inferred trees. All methods except SCITE removed the redundant inner nodes of the trees (i.e., nodes with not more than one child). For the SCITE method, to obtain a comparable small tree, we compressed the tree in the same manner.

In the trees inferred by BitPhylogeny, SCITE, SiFit, SASC, and SCIPhI (Fig 1 and Table 2), normal and cancer cells were not separated into distinct subtrees. In the inferred trees by SCIPhI, BitPhylogeny, and SCITE, normal and cancer cells were even assigned to the same clone, which seems unlikely as an evolutionary scenario. In contrast, the trees of OncoNEM, SiCloneFit, and Scelestial effectively separated normal and cancer cells. OncoNEM placed all the normal cells in one clone, while Scelestial and SiCloneFit separated all the cancer cells and normal cells into distinct subtrees. SCIPhI, OncoNEM, SCITE, and SASC trees contain cancer cells as ancestors of normal cells.

**Table 2:** Comparison of single-cell phylogenetic trees reconstruction methods on real single-cell genomic datasets.

	Muscle-invasive bladder tumor (Fig 1)				Metastatic colorectal cancer, Patient 1 (Fig 5)				Metastatic colorectal cancer, Patient 2 (Fig 6)			
	Mixed cancer and normal sample	Cancer sample as parent of normal sample	Cancer sample components	Cancer sample not in largest component	Mixed cancer and normal sample	Cancer sample as parent of normal sample	Cancer sample components	Cancer sample not in largest component	Mixed cancer and normal sample	Cancer sample as parent of normal sample	Cancer sample components	Cancer sample not in largest component
BitPhylogeny	18	**0**	13	37	67	6	7	34	129	10	8	34
Scelestial	**0**	**0**	**1**	**0**	**0**	**0**	2	2	**0**	**0**	**2**	**1**
OncoNEM	**0**	1	**1**	**0**	118	**0**	2	1	106	2	**2**	3
SASC	**0**	2	3	6	**0**	33	13	17	**0**	52	10	9
SCIPhI	23	1	4	22	16	**0**	6	12	128	2	5	29
SCITE	20	2	7	19	24	2	4	11	109	2	4	6
SiCloneFit	**0**	**0**	**1**	**0**	**0**	**0**	**1**	**0**	**0**	**0**	3	2
SiFit	**0**	**0**	3	6	**0**	**0**	9	43	**0**	**0**	7	10

Formal definition of the measures (columns) presented in Section F in S1 Text. In all the measures lower values represent better performance. The best performance of each measure for each dataset is shown in boldface.

The evolutionary trees (Fig 1) returned by all the algorithms except Scelestial were directed. The most plausible scenario for cancer evolution is the rooting of a cancer lineage close to this root or to some internal lineage of normal cells, instead of cancer cells being placed as ancestral to normal cells. SiCloneFit as well as SiFit did not place any cancer cells as ancestors of normal cells.

### 2.4 Case study 2: A single-cell dataset from metastatic colorectal cancer

We inferred lineage trees by all methods on single-cell data of colorectal cancer from two patients [25]. In this dataset, 178 single-cell samples were gathered from the first patient: 117 samples from normal cells, 33 samples from the primary tumor, and 28 samples from metastasis of the tumor to the liver. The data represent 16 genomic sites with 6.7% missing values directly taken from [25]. The single-cell data for this dataset was generated from single nuclei captured from patient samples and isolated by FACS and then barcoded using a 1000 cancer gene panel (T1000) and sequenced on the Illumina platform.

**Fig 5:**
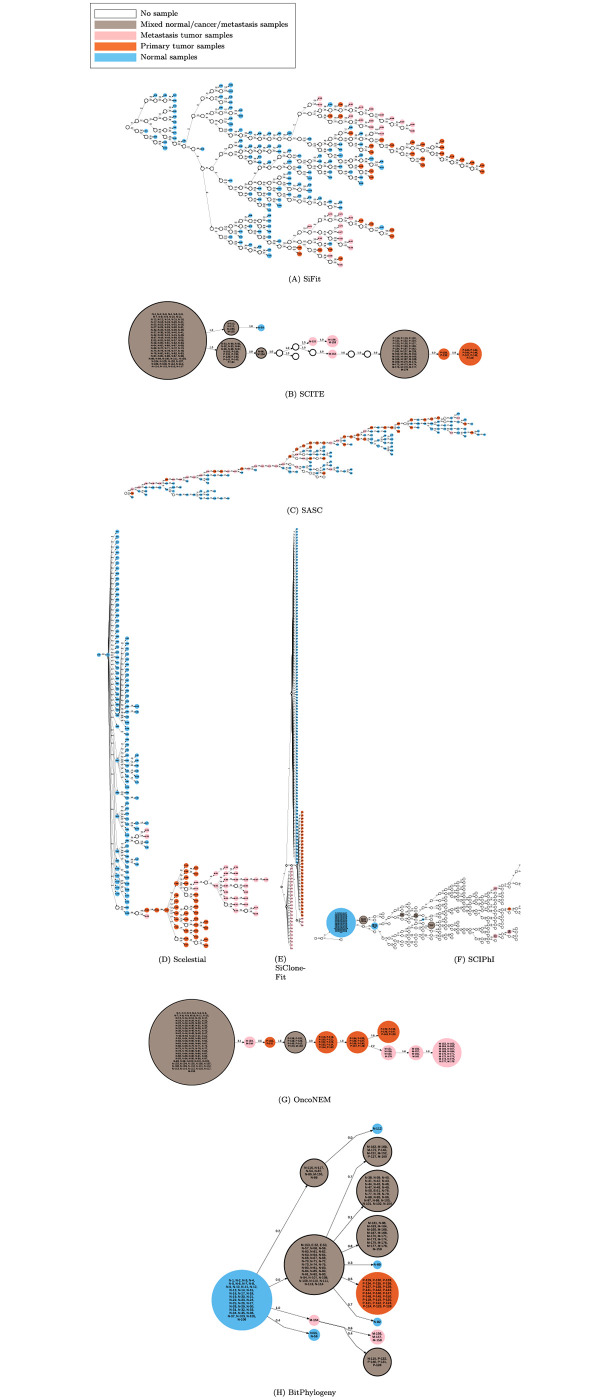
Lineage tree inferred by the different methods on a single-cell dataset from the first colorectal cancer patient.

In the reconstructed lineage trees for the first patient (Fig 5), OncoNEM, BitPhylogeny, SCIPhI, and SCITE suggest clones including both normal and cancer cells, which, as outlined above, is unrealistic. SASC produced a lineage tree with several normal and cancer cells that evolved from cancer clones. Removing normal cells and their direct parent from the SiFit tree (with the background assumption that normal cells produce normal cells) produced nine connected components, demonstrating the substantial mixing of cancer and normal cells. For this case, SiCloneFit separated all the cancer cell samples from the normal cells and metastatic cell samples well. However, SiCloneFit suggested metastatic cell samples to be closer to normal cell samples than the primary tumor cell samples in the inferred lineage tree. Scelestial placed two of the metastatic cell samples outside of the subtree of cancer cells, and one of metastatic cell samples outside of the subtree of the other metastatic samples. The comparison between SiCloneFit and Scelestial’s results shows that SiCloneFit produced a better partitioning of normal, primary tumor, and metastatic tumor cell samples. On the other hand, SiCloneFit produced a less resolved tree and also produced a tree with a not realistic evolutionary distance between normal, primary and metastatic cell samples. Scelestial on the one hand placed three metastatic cell samples not in a subtree of the rest of metastatic cell samples, but Scelestial inferred a tree with a better evolutionary placement of metastatic tumor cell samples relative to primary tumor cell samples.

From the second patient, 182 single-cell samples were taken: 114 normal cells, 29 primary tumor cells, and 39 metastatic cells. Thirty-six features were extracted by evaluation of the cancer mutations of the samples. Of all sites, 7.7% were missing values.

**Fig 6:**
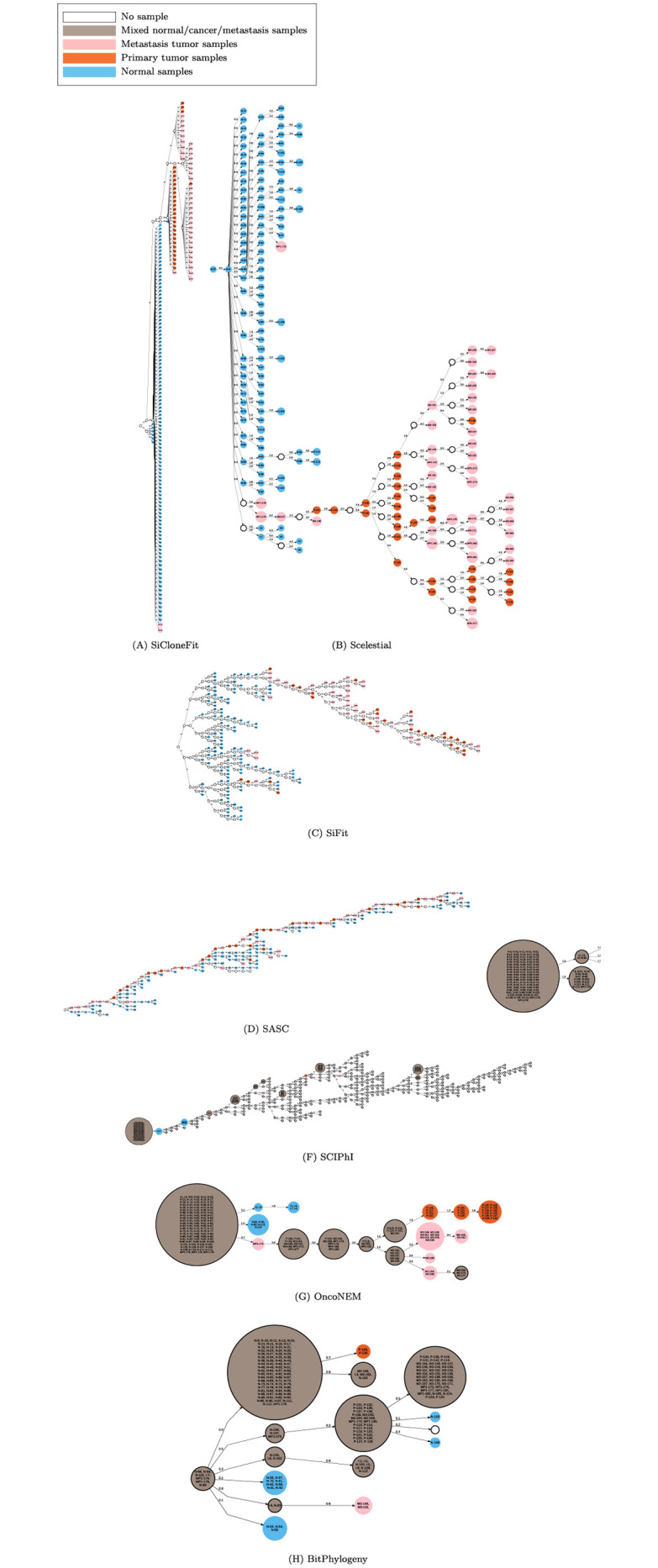
Lineage tree inferred by the different methods on a single-cell dataset from the second colorectal cancer patient.

All algorithms, except Scelestial, separated normal from cancer cells less well than for the first patient (Fig 6 and Table 2). Similar to their trees for the first patient, OncoNEM, BitPhylogeny, SCIPhI, and SCITE suggested several mixed clones including normal and cancer cells. SASC produced a lineage tree with several normal and cancer cells evolving from each other. SiFit produced a lineage tree with two subtrees containing both normal and cancer cells as well as three cancer samples outside these two subtrees. Except for one cell, all cancer cells were separated from normal cells into one subtree in Scelestial’s tree. In the SiCloneFit tree, the cancer cells MP1–176 and MP1–179 were misplaced outside of a cancer cell lineage and closer to normal cells. Metastatic and primary tumor cells were not well separated from one another in the trees generated by Scelestial as well as by SiCloneFit. Overall, though, Scelestial and SiCloneFit created the most realistic lineage trees of all the methods analyzed.

### 2.5 Mutation pattern analysis of Scelestial’s phylogeny for case studies

In analysis of the placement of normal cells as parents of cancer cells in the phylogenies, as in the case for single-cell case studies, placement of a normal cell like BN15 as the parent of BC18 in the first case study (Fig 1) suggests an evolutionary process in which a cell, which was not present in the sampled cells, was ancestral and very similar to the normal cell BN15, leading to a cancer cell lineage.

Except for missing values, all normal samples including BN15 have the same mutation pattern in the 443 loci identified in this study. Of the 443 loci, 21 were in 17 known cancer-related genes (*MTOR*, *PDE4DIP*, *PDE4DIP*, *PDE4DIP*, *TPR*, *SF3B1*, *RAP1GDS1*, *RAP1GDS1*, *EBF1*, *NSD1*, *RAD21*, *PSIP1*, *BMPR1A*, *NT5C2*, *ATM*, *CLIP1*, *MYH11*, *USP6*, *USP6*, *RABEP1*, and *BCR*) specified in Tier 1 (genes with documented cancer related activities) of the Cancer Gene Census database [27]. All normal cells and BC18 had mutations in 4 of these genes (*EBF1*, *NT5C2*, *ATM*, *RABEP1*), and either BC18 or BN15 had missing values in the remainder. Furthermore, BC18 and descendant cells had mutations in the cancer-related genes (in *PDE4DIP*, as well as a back mutation in *MTOR*, and *USP6*) in comparison to normal cells, which may have contributed to turning this lineage into an active cancer line.

In the second case study for the first patient (Section 2.4, Fig 5), the normal cell N-103 was placed as the parent of a big subtree with 26 cancer cells. Already some normal cells have several mutations in cancer-related genes listed in the COSMIC database [27], such as N-54 (a mutation in *TCF7L2*), N-67 (mutations in *TRRAP*, *TP53*), N-102 (a mutation in *KRAS*), N-103 (a mutation in *TP53*), and N-111 (a mutation in *GATA1*), and one unlisted one in *EYS*. The mutation in *TP53* was common in primary and metastatic tumor cells (54 mutated samples and 2 non-mutated and 4 missing values in 58 primary and metastatic cells). Thus the sample N-103 could be a precancerous sample having gained a mutation in *TP53*, which is a known tumor suppressor. Among most of the descendant cancer cells, more cancer-related changes were obtained, such as in *CCNE1*, *POU2AF1*, and *KRAS*.

In the phylogeny for the second patient (Section 2.4, Fig 6), the normal cell N-9 was placed at the ancestor of all cancer cells as well as normal cells, except for N-27. In the normal cells, in N-82, N-83, N-85, N-113, and N-114, loci in *CIITA* were different than in the cancer cells. Additionally, differences were observed in *NR3C2*, *ATR*, *ALK*, *EPHB6*, *SPEN* in intermediate cells (I-1 to I-8) relative to the cancer cells.

Excluding mutations appearing in intermediate or normal cells, we partitioned the remaining mutations in two sets based on their appearance in metastatic cells, denoted as M-mutations, the mutations appearing at most once in primary tumor cells (mutations in *LINGO2*, *IL7R*, *LINGO2*, *SPEN*, *F8*, *LAMB4*, *PIK3CG*, *LINGO2*, *PTPRD*, *FUS*, *NR4A3*, *HELZ*, *PRKCB*) and P-mutations (mutations in *APC*, *FHIT*, *ATP7B*, *LINGO2*, *LRP1B*, *CHN1*, *IL21R*, *APC*, *TOX*, *MN1*, *MYH11*, *TP53*, *NRAS*, *CDK4*, *LINGO2*, *STRN*). Of these, *APC*, *FHIT*, *LRP1B*, *IL21R*, *APC*, *MN1*, *MYH11*, *TP53*, *NRAS*, *CDK4*, and *STRN* from the P-mutations set, as well as *IL7R*, *SPEN*, *FUS*, *NR4A3* from the M-mutations set are known cancer related genes.

MP1–178 and MP1–179 have only 4 mutations from the P-mutations set, and were placed in Scelestial’s tree between the normal samples and cancer samples. Other descendant cancer cell samples, except for MP1–176, which was misplaced inside a normal lineage, have more P-mutations. Sample M3–147, the direct descendant of MP1–176 and ancestor of the remaining cancer samples in Scelestial’s tree, has all the mutations of MP1–179 as well as 4 more P-mutations. Thus, Scelestial’s tree represents a potential evolutionary scenario in which mutations in *APC*, *TOX*, *MN1*, and *TP53* were gained initially (by an ancestral cell similar to MP1–179), then a descendant gained mutations in ATP7B, IL21R, NRAS, LINGO2 and the rest of cancer cells descended from this cell.

Details of analysis of the placement of cancer cell samples close to normal cells for all three real single-cell datasets are given in Section I in S1 Text.

### 2.6 Run time efficiency

**Fig 7:**
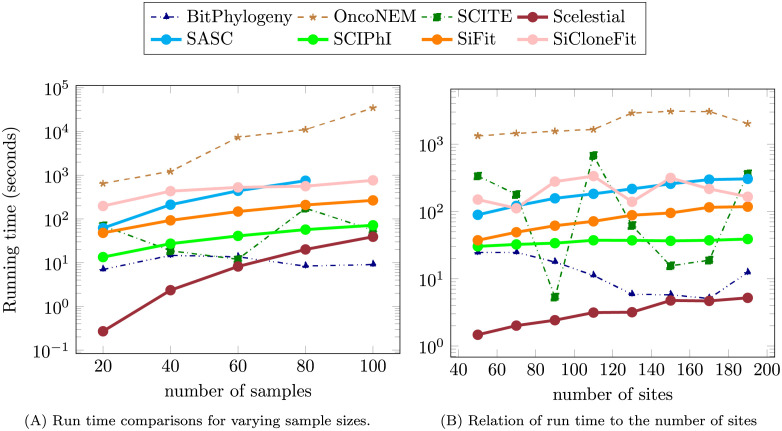
Run time comparison in relation to the number of samples and sites.

We compared the run times of the eight methods with default settings (as described in Section 2.1) on the 110 datasets generated by the OncoNEM simulator (Fig 7). We simulated data with 10 mutations on average at each evolutionary step, a range of 20 to 100 samples, and a range of 50 to 200 sites. The runtime comparisons were performed on virtual servers with 24 to 69 Intel QEMU Virtual 2.8 GHz core CPUs (version 2.1.2), and 100 to 3000 GB RAM. To disregard the effect of using several cores, the user time was considered as the actual time of a task.

SCITE, SCIPhI, BitPhylogeny, and Scelestial were the only methods finishing their task across the whole range of sample sizes within 100 seconds. Scelestial, SCIPhI, and SCITE’s run times grew almost linearly with an increasing number of samples. The run time of BitPhylogeny does not seem to be directly related to the number of samples but was random for the tested cases, since it applies MCMC and its run time depends on the number of iterations. When we increased the number of sites, the run time of Scelestial also grew almost linearly. Next to Scelestial, BitPhylogeny and SCITE were the fastest methods. Over all cases, Scelestial was faster than all other methods in seven cases; BitPhylogeny was the fastest in six cases.

**Fig 8:**
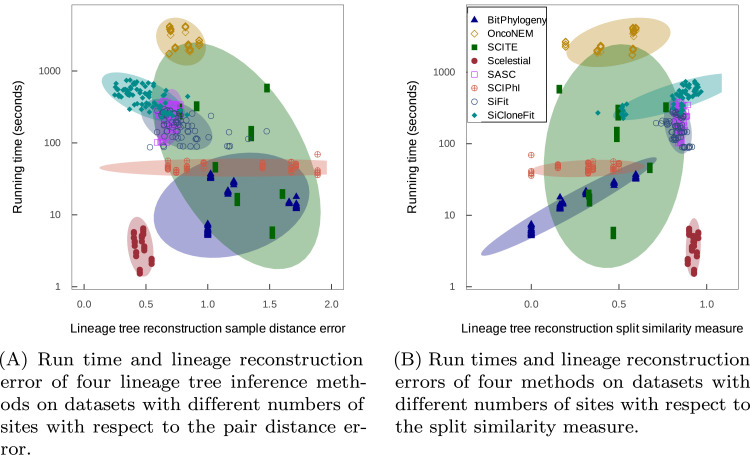
Comparison of methods with respect to running time and lineage tree reconstruction error. For the results obtained from different execution of a method a confidence ellipsoid is calculated and shown as the shaded area around the resulting points. The confidence interval is calculated under the assumption of normal distribution of the points in the two dimensional diagram.

With these data, as well as being fastest, Scelestial, as before, also had the smallest average error in lineage tree reconstruction (Fig 8A). Among 74 cases, in terms of sample distance error, Scelestial performed best in 40 cases and SiCloneFit in 34 cases. In terms of the split similarity measure of topological correctness, Scelestial performed best in 64 cases and SiCloneFit in 11 cases. Thus, in these tests Scelestial was best overall in both run time and error. SCITE and BitPhylogeny had similar split similarity, with SCITE being faster than BitPhylogeny on average. Considering the pair distance error, the performance of BitPhylogeny was better than that of SCITE. The performance of OncoNEM with respect to the pair distance error was better than that of BitPhylogeny and SCITE in a lot of cases and on average. However, the variance of the pair distance error and the split similarity of OncoNEM’s results were higher than those of others. The range of variation of performance for OncoNEM is clearly shown in the split similarity measure chart for this dataset (Fig 8B).

According to theoretical analysis, the run time of Scelestial is polynomial with respect to the number of samples with exponent *k*, which is a parameter for the Steiner tree approximation algorithm. In Fig 7, the quadratic form of the run time might not be directly evident. This is normal because the chart is cropped for large values to show the differences for all the algorithms except OncoNEM and SiFit. The chart is also drawn logarithmically in the y-axis, which makes it hard to see the actual growth in it. SCIPhI, SCITE, and BitPhylogeny show similar behavior. In practice, the run times of all the algorithms except for Scelestial and SCIPhI grew substantially with an increasing number of sites.

On large datasets, both in terms of the number of sites and the number of samples, Scelestial and SiCloneFit perform best with respect to the split similarity measure and lineage tree reconstruction error (Figs C and D in S1 Text). Similar to the same observation for normal-sized samples (Fig 8), Scelestial’s results have less variance than those of SiCloneFit. Note that OncoNEM has errors for datasets with high numbers of samples.

SASC, SiCloneFit, SCITE have a longer running time than SCIPhI, BitPhylogeny, and Scelestial (Panel (II) of Fig E in S1 Text). Of these three fast methods, Scelestial performs best both in terms of split similarity and lineage tree reconstruction error measures (Fig C in S1 Text). On datasets with large numbers of samples, the two methods with the highest accuracy, Scelestial and SiCloneFit, in addition to SASC, are the slowest ones. In this range of samples (500–900), the running time of Scelestial greatly increased, and at 800 samples, Scelestial became slower than SiCloneFit (Panel (I) of Fig E in S1 Text). The accuracy of the methods is similar to their accuracy on data sets with a larger number of sites (Fig D in S1 Text), i. e. Scelestial and SiCloneFit perform best with respect to the lineage tree reconstruction error and sample distance measures. In conclusion, considering accuracy and run time, Scelestial and SiCloneFit performed best across different data sets. Although the two methods show similar accuracy, on datasets with many sites, Scelestial is faster, and on datasets with many samples (800 samples or more), SiCloneFit is the faster option. Note that in comparison with SiCloneFit, Scelestial shows less variation across performance measures on different datasets.

## 3 Materials and methods

### 3.1 Data format

We model the data as an *m* by *n* matrix *D* with single-cell samples as columns and features as rows. The element of *D* corresponding to a single-cell *c* and a locus *f* is denoted *D*[*c*, *f*] and represents the result of a variant call obtained from a single-cell sample of a diploid genome, which may be one of the 10 states from the set {*A*/*A*, *T*/*T*, *C*/*C*, *G*/*G*, *A*/*T*, *A*/*C*, *A*/*G*, *T*/*C*, *T*/*G*, *C*/*G*} or a missing value *X*/*X*. When the data do not provide all the information (e.g., for data obtained from OncoNEM’s simulation tools), we can convert 0/1 (reference state/variant state) matrices to the 10-state format by coding 0 as A/A and 1 as C/C. All the single-cell lineage tree reconstruction methods we consider here support missing values in their input matrices. With this coding, we cannot differentiate between the case of two similar alleles, e.g. A/A and the case of a missing allele in one strand. We hope that considering an error as a regularization method helps us to find the model (which is a lineage tree) that fits the data best.

### 3.2 Synthetic data generation via tumor simulation

We developed a tumor growth simulation method as a data source for the evaluation of Scelestial and for a comparison with state-of-the-art methods. The simulation has three phases: (1) simulation of evolution, (2) sampling from the tree, and (3) simulation of sequencing.

In the simulation of evolution phase, the evolutionary process is simulated and a tree is generated. This simulation is based on evolutionary events that happen in a tumor. We modeled the cell division, mutation, and selection in the evolutionary process of the tumor in this simulation. Each node in the evolutionary tree represents a cell (or representative of a bulk of similar cells) in the history of tumor formation. To each of these, an advantage value is assigned that shows the relative growth or division advantage of the cells corresponding to that node. The advantage value of a node is the same as its parent advantage value plus or minus a uniform random number. The evolutionary tree is constructed through several steps. In each step, one new node is generated and its parent is chosen from the current nodes with a probability proportional to their advantage values. We calculated the advantage value for the newly born node as a random perturbation added to its parent’s advantage value. The actual sequence for the new node is calculated from the sequence of the parent with some random mutations. The number of mutations from its parent is derived from a Poisson distribution about an average parameter specified in the input. The locations of the mutations are chosen uniformly from all the loci.

In the sampling phase, samples are chosen from the nodes of the tree with a probability which is proportional to their advantage values. In the simulation of the sequencing phase, missing values and errors are then incorporated by stochastic processes.

### 3.3 Synthetic data generated by OncoNEM’s simulation tool

We used simulated data generated by OncoNEM’s simulation software for our evaluation. The OncoNEM simulator is based on the evolution of clones. First, it generates a lineage tree, then it assigns mutations to tree nodes under the infinite site assumption. Afterwards, it generates output sequences by sampling from the tree and generating sequences with single-cell sequencing issues, including missing values, false positives, and false negatives.

### 3.4 Comparison of a resulting lineage tree with a ground truth

We define two measures between trees: (1) a distance measure that compares distances between samples in two trees, and (2) a similarity measure that compares the set of splits made by two trees applied to the set of samples. For both measures, we used a weighted version of classic 0/1 measures.

Different measures used in the literature are clustering accuracy [12], order of mutation in a phylogenetic tree, ancestor-descendant accuracy of mutations, different lineages, and co-clustering mutations [12]. These measures are used for evaluating mutation trees, which is not what we generate in the Scelestial method.

#### 3.4.1 Sample distance measure between trees

We quantify the distance between two trees based on the shortest-path matrix proposed by [5] and the path-length-difference metric [28]. The basic idea is that we calculate pairwise distances between each pair of input cells in a tree *T*. We also create a pairwise distance matrix *PD*_*T*_ between the inputs for the tree. Following this, we normalize the matrix *PD*_*T*_ to obtain ${\bar{PD}}_{T}$ as ${\bar{PD}}_{T}\left[i,j\right]=PD_{T}\left[i,j\right]/\sum_{x,y}PD_{T}\left[x,y\right]$. Since different concepts of weight for the tree edges are used in different methods, the normalization phase allows us to neglect absolute values and only consider the relative edge distances in the lineage trees provided. We define the distance between two trees *T* and *T*′ as $D\left(T,T^{\prime }\right)≔\sum_{i,j}\left|{\bar{PD}}_{T}\left[i,j\right]-{\bar{PD}}_{T^{\prime }}\left[i,j\right]\right|$, which represents the distance between the normalized pairwise distance matrices for the two trees. The value of *D*(*T*, *T*′) lies in the range between 0 and 2.

#### 3.4.2 Split similarity measure between trees

We defined the split similarity between two trees *T* and *T*′ as the similarity between two sets of splits generated by two trees. For each edge *e* in a tree *T*, we define the split *S*_*e*_ as the set {*A*, *B*}, where *A* and *B* are the set of samples separated by the edge *e* in *T*. We define a similarity between two splits {*A*_1_, *B*_1_} and {*A*_2_, *B*_2_} as the number of samples that are split similarly in two splits. However, since there is no difference between *A* and *B* in two splits, we define the similarity score as:

$$\text{max}\{|A_{1}\cap A_{2}|+|B_{1}\cap B_{2}|,|A_{1}\cap B_{2}|+|B_{1}\cap A_{2}|\}$$

To calculate the distance between two sets of splits, we find the mapping between the elements of two sets with the maximum similarity score. The mapping similarity score is the sum of the similarity of the scores of matched splits. We then defined *D*_*S*_(*T*, *T*′) as the normalized matching score which is the mapping score between *T* and *T*′ divided by the mapping score of *T* with *T*. The split score value is between 0 and 1. The split similarity measure is a relaxed version of the symmetric difference or partition metric as in [29]. For each edge of a tree two splits are defined as the subset of samples that are located on one side of the edge. The set of all these splits is called the split set. The distances calculate a measure given two sets of splits, The partition metric for two split sets counts the number of common sets in two split sets. We calculate a relaxed version of it: for each two splits, we calculate a similarity measure between those as the size of their intersection, and find a matching between splits included in the split sets with the maximum sum of split-similarity scores. So, we use some rational similarity between the splits, as an extension to the approach presented by [29], which uses 0/1 scores between two splits.

### 3.5 The Scelestial algorithm

The Scelestial algorithm is a maximum parsimony algorithm, in other words, its objective function is to find an evolutionary tree for a set of sequenced samples that minimize the number of changes (mutations). It is shown by Alon et al. [23] that under the Neyman 2-state substitution model [30], which is suitable for cell lineages that evolve on short time scales, the maximum likelihood tree inference problem could be approximately reformulated as a maximum parsimony problem. Thus, the Scelestial algorithm tries to find a solution for a maximum likelihood problem via approximately solving a maximum parsimony problem.

In the Neyman 2-state substitution model, sequences are modeled as two state characters. The loci mutate independently and to each edge *e* of the evolutionary tree a mutation probability of *p*_*e*_ is assigned that represents this mutation probability. The maximum likelihood inference problem then would be the problem of finding an evolutionary tree, mutation probabilities, and internal nodes’ sequences that maximize the likelihood function. Alon et al. [23] show that the maximum likelihood problem could be approximately reformulated as a maximum parsimony problem. At last, the reformulated maximum parsimony problem is solved approximately via an approximation algorithm for the Steiner tree problem.

We incorporate an approximation algorithm for this problem provided by Berman et al. [24] and its modification for lineage tree reconstruction [23]. We modified the algorithm to support missing values. In the following, we examine the details of the Steiner tree problem, the reduction of lineage tree reconstruction to the Steiner tree problem and the incorporation of missing values into our method. Although the theoretical approximation results could only be applied for the two state case without the missing values, we can extend the algorithm to be used for these cases.

**Fig 9:**
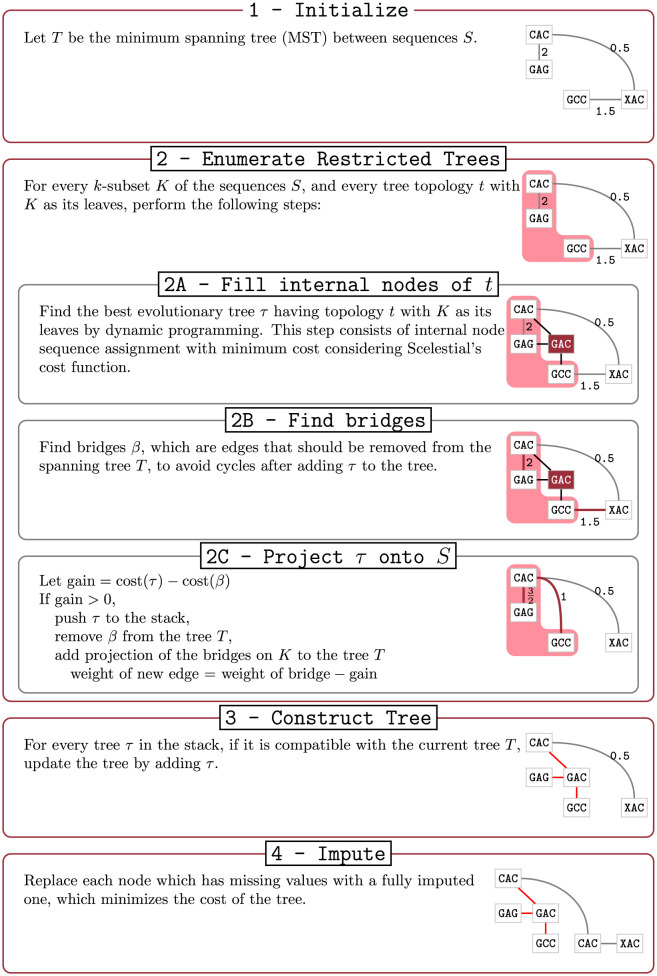
The details of the Scelestial algorithm. The inputs to the Scelestial algorithm are a) a set of sequences *S*, b) the degree of restriction of the restricted Steiner tree *k*. The value *k* represents the size of trees to be considered as potential improvement for the tree *T* (details in Section 3.5.1). An example is shown at the right side of each step. The input sequences *S* are “CAC”, “GAG”, “GCC”, and “XAC” (“X” represents a missing value). In step 1 the tree *T* is initialized with the minimum spanning tree of the input sequences *S*. The edge lengths represent the cost of the edge according to the Scelestial’s cost function (see Section 3.5.5). In step 2 an example of a subset of sequences for *K* is highlighted in the picture. In step 2A a tree *τ* over leaf nodes *K* is shown. In step 2B bridges are highlighted as red edges between input sequences. In step 2C the result of adding tree *τ* to the current tree *T* is shown. In this example the bridges shown in step 2B are removed and two edges between nodes *K* corresponding to bridges are added. For each bridge an edge between the two nodes from *K* which their path passed through the edge is added. The new costs are defined as it is shown in step 2C. In step 3, the trees *τ* are added to the tree with their corresponding internal nodes. In step 4 lowest cost imputation of sample nodes are added to the tree, in the sample place as input sequences.

A schematic view of the Scelestial algorithm is provided in Fig 9. The main part of the Scelestial algorithm is described in Algorithm 1. Sub-modules of the Scelestial algorithm are described in Algorithm 2–5. The inputs to the algorithm are the sample sequences, and the algorithm infers the phylogeny as well as its internal nodes.

The Berman approximation algorithm for the Steiner tree problem and theoretical analysis was provided by Berman, and the suggestion of using this approximation algorithm for Neyman 2-state sequences was made by Alon. In this work, we implemented the algorithm, adapted it to be used for sequences with missing values and more than two states. Note that we are not aware of analytically proven guarantees regarding the performance of the theoretical algorithm for more than two-state sequences.

#### 3.5.1 The Steiner tree problem

The Scelestial algorithm is based on the Berman approximation algorithm for the Steiner tree problem [24]. The input of a Steiner tree problem consists of a weighted graph *G* = (*V*, *E*, *w*) and a subset of its vertices *S* ⊆ *V*, which are called terminals. It is convenient to suppose that the weight function *w* satisfies the triangle inequality (i.e. *w*(*x*, *y*) + *w*(*y*, *z*) ≤ *w*(*x*, *z*), for all three vertices *x*, *z*, *y* ∈ *V*). In the case of no missing values occurring in the data, the triangle inequality constraint is satisfied, for example, for the Hamming distance between nodes. However, in the case of single-cell data, which contain a lot of missing values, we should consider this constraint carefully.

The Steiner tree problem is the problem of finding a minimum-weight connected subgraph of *G* that contains all the terminals *S*. The Steiner tree problem is an NP-hard problem and it is known that under reasonable complexity assumptions, no approximation algorithm can approximate the result better than a factor of 96/95 ≈ 1.0105 [31].

Most approximation algorithms for the Steiner tree problem focus on results that consist of a set of subtrees, each having *k* terminals at most, for some constant *k*. A solution for the Steiner tree problem with this property is called a *k*-restricted Steiner tree. We call the value *k* the restriction number of a Steiner tree. Borchers and Du [32] showed that restriction of the search space to *k*-restricted Steiner trees does not change the approximation factor of an algorithm too much. More specifically, if *k* = 2^*r*^ + *s* where 0 ≤ *s* ≤ 2^*r*^, the restriction to *k*-restricted Steiner trees reduces the approximation ratio to

$$\frac{r2^{r}+s}{\left(r+1\right)2^{r}+s}$$

#### 3.5.2 Berman’s approximation algorithm for the Steiner tree problem

The Berman et al.’s [24] approximation algorithm consists of three phases: examination, evaluation, and application.

During the examination phase, the algorithm maintains a minimum spanning tree *M* on the terminal set *S*. In this phase, the algorithm considers all the subsets *K* of the terminals with a size of at most *k* (for some fixed constant *k*) and all the topologies *τ* for the trees with *K* as its leaves. For each *K* and *τ*, the algorithm finds the best lineage tree *t* (i.e., the best sequences for the internal nodes). This could be done through dynamic programming. By adding *t* to the spanning tree, *M* will create (*k* − 1) cycles. The algorithm finds a subset of edges of *M* with maximum cost to be removed from *M* + *t* to obtain a tree again. These edges are called bridges *β*. A value gain is defined for *t*, which is equal to the amount of decrease in the resulting tree if we decide to incorporate *t*, which is equal to cost(*β*) − cost(*t*), where the cost of a set of edges is defined as the sum of the costs of its elements. If the the gain is greater than 1, the algorithm adds *t* to a stack for the evaluation phase, it removes *β* from *M*, and adds some new edges to *M* instead. For each edge *e* of *β*, the algorithm finds the vertices *u* and *v* from *K* which are going to be disconnected after removing *e*. It adds the edge (*v*, *u*) to *M* with the cost cost(*e*) − gain. At the end of the examination phase, we will have a stack of some trees and *M*.

In the evaluation phase, the algorithm pops the trees *t* one by one from the stack. It starts with an initially empty set of edges *M*′. At each step it checks if *t* does not make a loop with *M*′. If this is the case, the algorithm accepts *t*; otherwise, it rejects *t*. Finally, in the application phase, the algorithm merges the accepted trees.

The run time of the algorithm for a general Steiner tree problem is $O(n^{3}+n^{k+\frac{1}{2}}+N^{k-2}n^{k-1})$, where *N* is the number of vertices in the graph. Note that *n* is the number of terminal vertices *S*. The algorithm is an 11/6-approximation algorithm for the Steiner tree problem, if we set *k* = 3. If we set *k* = 4, the algorithm would be a 16/9-approximation algorithm. For larger values of *k*, we do not know a better approximation factor for the result of the algorithm, but the results of the execution of the algorithm show that the performance of the algorithm gets better as we increase the value of *k* [24].

#### 3.5.3 Modeling lineage tree reconstruction as a Steiner tree problem

The single-cell lineage tree inference problem can be modeled in the following way. Let *g*_*i*_ for 1 ≤ *i* ≤ *n* be the set of input sequences over some alphabet Σ + *μ*, where *μ* is a special character representing the missing value. Each location on a sequence could be considered as a feature, which may be a locus in the sequenced genomes or may represent a genomic aberration. We suppose that all the sequences have the same length *m*. A cost function is defined for any potential lineage trees to show how well a lineage tree is fitted to the data. Normally, the cost function assigns a cost to each edge of the tree, and the cost of a tree is the sum of its edge costs. The lineage tree inference problem is the problem of finding a minimal cost lineage trees that contain all the input sequences *g*_*i*_ and potentially some other nodes. In the resulting tree, all non-input nodes are assigned a label of length *m* from the alphabet Σ.

A general lineage tree inference problem that does not incorporate missing values could be modeled as a Steiner tree problem as follows. Let *G* = (*V*, *E*, *w*) be the graph containing all sequences of length *m* from the alphabet Σ that have edge weights derived from the tree cost function. Thus, the lineage tree problem would be equivalent to finding a Steiner tree in this graph with the input sequences as its terminal set *S*.

#### 3.5.4 Incorporating an approximation algorithm for lineage tree reconstruction

We showed how lineage tree reconstruction could be modeled as a Steiner tree problem in the previous section. However, through this method, the size of the graph is |Σ|^*m*^, which is exponential to the length of input of the lineage tree reconstruction problem. On the other hand, some approximation algorithms do not consider all the vertices of graph *G* and they only work with the best Steiner trees over certain subsets of terminals. Since we can solve the Steiner tree problem over a small subset of terminals, we can use these approximation approaches.

To apply Berman et al.’s algorithm for lineage tree reconstruction, as already suggested by Alon et al. [23], for every subset *S*′ ⊆ *S*, with a of size at most *k*, we consider all the possible tree topologies with *k* leaves at most. Since the number *k* is constant, the number of these tree topologies would also be constant. Next, for the pair consisting of a subset *S*′ and a tree *T*, we find the minimum-cost Steiner tree by dynamic programming. The rest is done by the Berman [24] algorithm. This approach gives us an 11/6-approximation algorithm for the lineage tree reconstruction problem with *k* = 3 and a 16/9-approximation algorithm for *k* = 4. The cost of an edge between two nodes with the sequences *g*_*i*_[*t*] and *g*_*j*_[*t*] for 1 ≤ *t* ≤ *m* is ∑_*t*_ *c*(*g*_*i*_[*t*], *g*_*j*_[*t*]), for some cost function *c*. In this work, we assign a cost of 1 to every mutation and a cost of zero to non-mutated locations (i.e., *c*(*x*, *x*) = 0 for *x* ∈ Σ and *c*(*x*, *y*) = 1 for *x* ≠ *y* ∈ Σ).

#### 3.5.5 Support for missing values

To adapt the method to the single-cell setting, we incorporate missing values into the algorithm. We can simply do this by extending the domain of the character cost function $c:\Sigma \times \Sigma \to R^{*}$ to $c:\Sigma +\mu \times \Sigma +\mu \to R^{*}$. To do so, we should define *c*(*μ*, *x*) = *c*(*x*, *μ*) for *x* ∈ Σ as well as *c*(*μ*, *μ*); *c*(*μ*, *x*) could be considered as the cost of imputation for a locus. One may consider the assignment of *c*(*μ*, *x*) = *c*(*μ*, *μ*) = 0. However, this assignment violates the triangle inequality for the graph *G*. To preserve the triangle inequality between the edges’ weights, we may assign *c*(*μ*, *x*) = 0.5 + *ϵ* for some small constant *ϵ* (e.g., 10^−5^). Furthermore, letting *c*(*μ*, *μ*) = 0 does not violate the triangle inequality any longer.

#### 3.5.6 Missing value imputation

The original approach of representing lineage tree reconstruction as a Steiner tree problem does not consider the case of missing values in the input sequences. However, for single-cell data, this is important. We therefore designed a dynamic program that finds internal node sequences with non-missing values. As a result, missing values may occur only in input sequences. The translation of the missing value imputation task in our model would be the task of filling missing values with some characters from the alphabet Σ to minimize the cost function. To impute missing values after finding the lineage tree, we replace each missing value in a node with the most abundant character found within its neighbors.




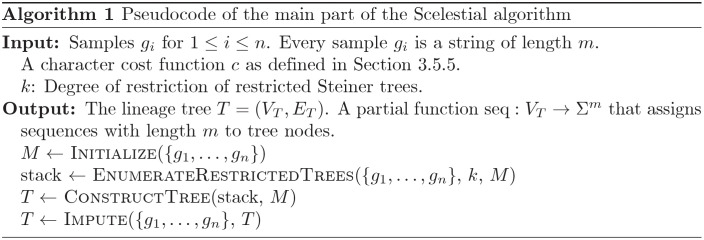







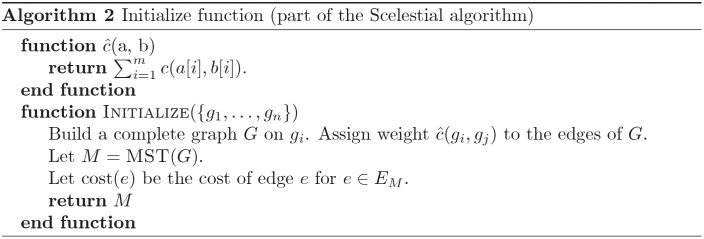







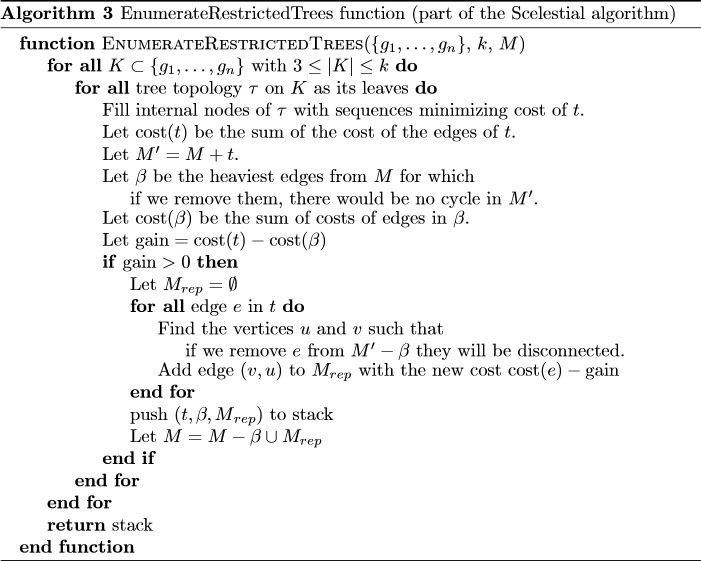







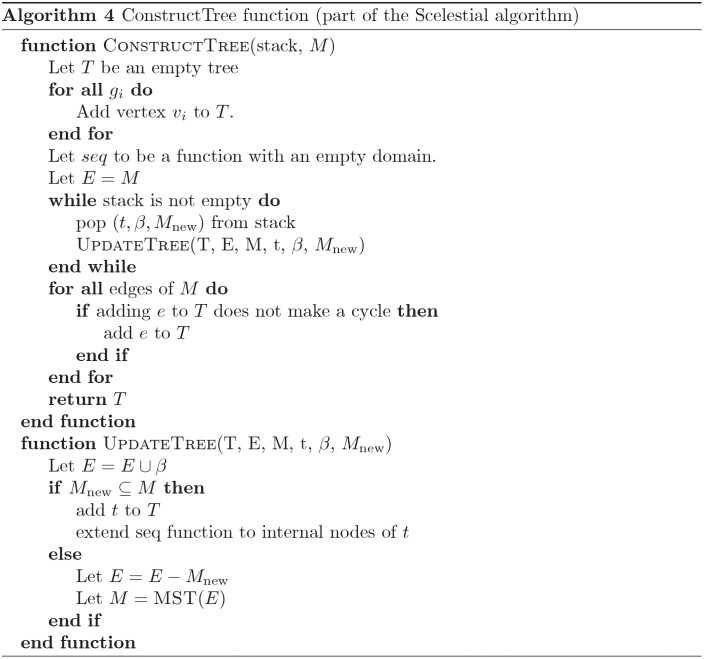







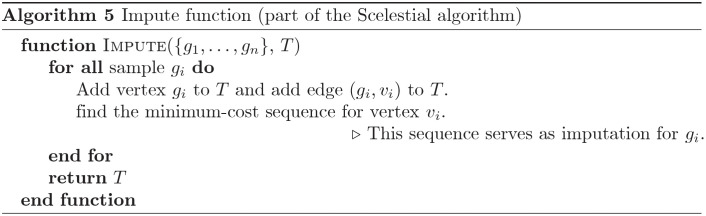




## 4 Discussion and conclusions

Inference of a tumor’s evolutionary history is a crucial step towards understanding of the common patterns of cancer evolution. There are a range of methods available for phylogenetic inference with single-cell datasets. For optimal performance, many of these probabilistic approaches require substantial time for optimization (e.g., using MCMC). The increasing sample sizes of single-cell datasets emphasizes the need for fast and scalable methods in this domain that also maintain high accuracy [33].

Here, we describe Scelestial, a computationally lightweight and accurate method for lineage tree reconstruction from single-cell variant calls. The method is based on a Steiner tree algorithm with a known approximation guarantee, which we adapted to lineage tree reconstruction for single-cell data with missing values by using a variation of the Steiner tree problem called the group Steiner tree. This problem is a special case of the group Steiner problem, in which the groups are sub-hypercubes of the original graph. To obtain a fast algorithm, we further adapted the group Steiner tree algorithm through modeling a hypercube corresponding to the missing values with one representative vertex. To facilitate the use of Scelestial, we provide an implementation as a freely available R package. From another point of view, Scelestial could be considered as a generalization of the neighbor-joining method. At each step, the neighbor-joining method finds the two most similar elements (samples) and merges them to build a tree. A generalization of this idea may consider more than two samples for merging in each step. Determining a good objective function for ranking three sample candidates is not trivial. Scelestial, based on Berman’s algorithm [24], provides a generalized guaranteed neighbor-joining method.

We evaluated Scelestial’s performance with a diverse set of test cases similar to modern single-cell datasets. The datasets were generated with various ranges of clones, false positives, false negatives, and missing values, all of which were derived from real single-cell datasets [25, 34]. The simulated data were produced by a tumor simulator emulating the process of tumor growth via mutation and proliferation. In this way, data for different tumors with various parameters were generated to assess computational methods over a wide range of data types. To confirm that the results on these simulated datasets are in line with results on simulated data commonly used in the field, we also incorporated evaluation on data sets generated with the OncoNEM simulator [5].

On these benchmark datasets, we compared Scelestial with seven other state of the art phylogeny reconstruction methods, namely BitPhylogeny [4], OncoNEM [5], SCITE [6], SASC [8], SCIPhI [10], SiFit [7], and SiCloneFit [11]. As the comparisons were not straightforward, we excluded B-SCITE [12], which uses a combination of single-cell and bulk data, as well as the approach of Kim and Simons [3], where the input is hard-coded, and PhISCS [13], as it does not infer a lineage tree. Of the methods using the k-Dollo assumption, namely SASC [8], which is based on simulated annealing, and SPhyR [9], which is based on integer programming, we included SASC in the comparison. For the assessment of lineage tree quality, we applied two commonly used metrics from population genetics and phylogenetics. In this comparison, Scelestial performed best at reconstructing the ground truth tree’s topology and also the similarity of the inferred to the ground truth tree when branch lengths were considered.

When the methods were applied to real single-cell cancer data, only Scelestial and SiCloneFit inferred lineage trees that separated all cancer cells from normal cells for all three datasets, except for a few cells. Among the other methods, OncoNEM was the only other method that did not mix cancer and normal cells in one single clone, although it mixed cancer and normal cells in the evolutionary tree. The run time analysis showed Scelestial to perform 4.5 times faster than the other methods on average when the default settings were used. Scelestial performs 24.6 times faster than all the other algorithms in some cases. This is particularly important, as the number of single-cell genomes published in individual studies continues to be on the rise, as are multi-dataset meta-studies, as in [1].

The Scelestial algorithm does not include a specific mechanism to handle irreversible evolutionary events such as deletions. Consequently, Scelestial is not suitable for datasets with frequent deletions, such as advanced metastatic tumors. On the other hand, Scelestial could be considered as a maximum parsimony method, and any evolutionary event matrix could be given to it as input. It could likely be adapted in practice to handle deletions by including an asymmetric matrix with very high weights for reversing such events. Notably, the real datasets analyzed in this manuscript (Sections 2.3 and 2.4), especially the metastatic colorectal cancer dataset, were solid tumor cases. The assessment of Scelestial and other methods on these datasets showed that Scelestial’s performance on these real datasets was generally similar to the best performing other method, which was SiCloneFit, demonstrating that this property is not essential for high quality tree inference with Scelestial from such data. In robustness analyses, Scelestial showed good performance for missing value rates less than 40% (Figs 3 and 4). On the computational side, Scelestial performed the calculation for 500 samples and 500 sites in less than 24 hours on standard hardware. Thus data generated by single-cell sequencing techniques, such as MDA, LIANTI, and MALBAC, and biological samples within these parameter ranges can straightforwardly be analyzed using Scelestial. On the other hand, Scelestial is not a good option for analyzing the data of low genome coverage methods such as DOP-PCR and DLP.

Overall, Scelestial substantially improves lineage tree reconstruction from single-cell variant calls across key criteria such as the scalability, run times, and accuracy of lineage tree reconstruction on both real and simulated data. Cell lineage reconstructions based on diverse tumor datasets in combination with massive data gathering, resulting from fast advancing technologies, provide a better understanding of the evolutionary landscape and the associated mutations of tumors, as may also indicate the dependencies between them [35–37]. These factors may make Scelestial instrumental in furthering our understanding of the mutational landscape and the mechanisms of cancer formation and survival, as omics technologies continue to thrive. Furthermore, the results of this paper can be seen as a case study for translating concepts from theoretical computer science into advances in computational biology.

A future direction of improvement over Scelestial could be adding support for asymmetric cost functions, such as the Dollo evolutionary model used by SASC and SPhyR. The core of Scelestial consists of iterative improvements via considering maximum parsimony trees for all k-subsets of samples. Considering a k-subset of samples, the maximum parsimony tree is inherently undirected and unrooted. To support an asymmetric cost matrix, the core of Scelestial could be changed by adding an outgroup to the tree and considering that in construction of all the k-subsets maximum parsimony trees. For this, further consideration will have to be given to the problem of merging k-subset trees and making a consensus tree, which could be a direction for future improvement.

## Supporting information

S1 Text Supplementary experiments and figures. (PDF)
