# Supplementary material for: Scelestial: Fast and accurate single-cell lineage tree inference based on a Steiner tree approximation algorithm
Source: PLoS Comput Biol. 2022 Aug 11;18(8):e1009100. doi: 10.1371/journal.pcbi.1009100 (PMC9426887; doi:10.1371/journal.pcbi.1009100)
Supplement: S1 Text — (PDF) [file pcbi.1009100.s001.pdf]

S1 Supplementary experiments and figures

Scelestial: fast and accurate single-cell lineage tree inference based on a Steiner tree approximation algorithm

Mohammad-Hadi Foroughmand-Araabi[1], Sama Goliaei[1], Alice C. McHardy[1,*]

1 Department of Computational Biology of Infection Research, Helmholtz Centre for Infection Research, Braunschweig, Germany

* Corresponding author (amc14@helmholtz-hzi.de)

A Comparison of methods on the simulated data

Fig A represents an extension of Fig 2 of the main manuscript including the error bars.

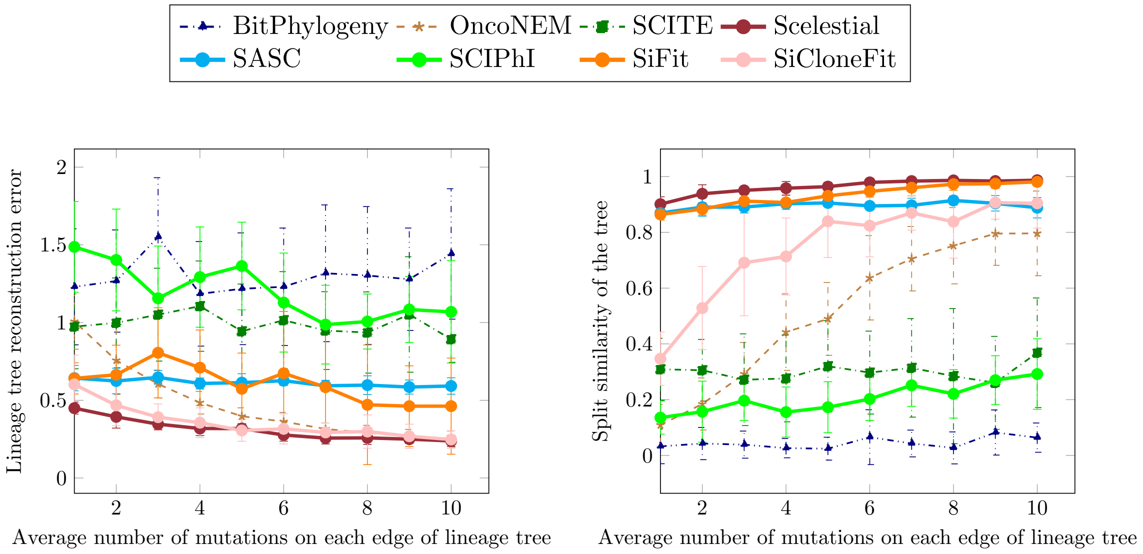

((I)) Sample distance measure

((II)) Split similarity measure

Fig A: Comparison of the methods for single-cell lineage tree reconstruction on simulated tumor data.
Note that in case of the lineage tree reconstruction error (Fig A(I)), lower values show a better reconstruction. On the other hand, the split similarity measure represents (Fig A(II)) similarity between the reconstructed tree and the ground truth tree, making higher values favorable.

B Evaluation of imputation by Scelestial

The Scelestial method does not impute the missing values. However, we can define an imputation for missing information by assigning characters that minimize the cost function defined in the Scelestial method. We developed this imputation and, using this, evaluated the number of errors in an imputation for Scelestial using a dataset generated by OncoNEM simulation (Section 2.6 of the main manuscript, Fig B). On average, 76% of the sites were imputed correctly, and there was little variance with changing the number of samples over the range of simulation parameters used in this experiment (Fig B(II)).

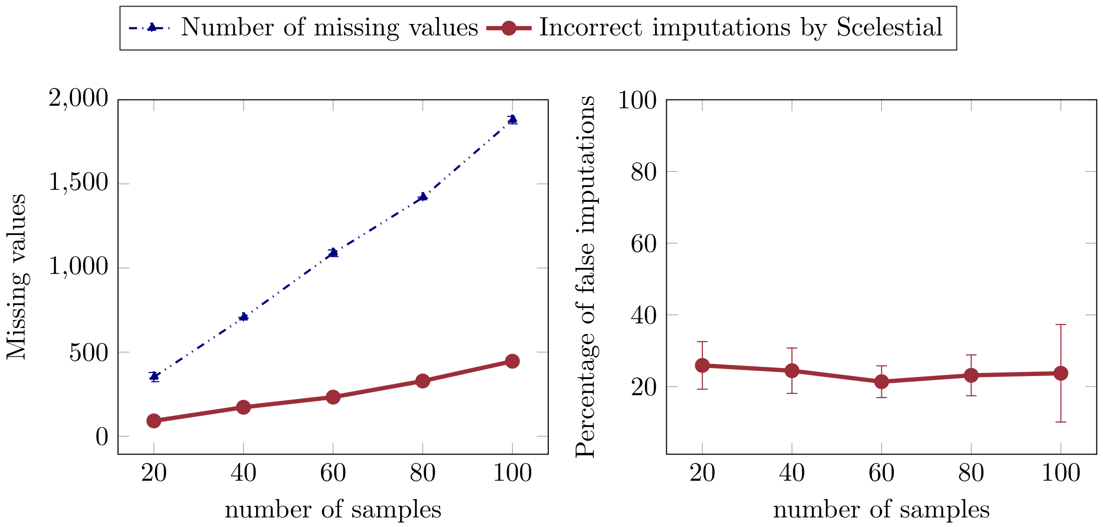

((I)) Imputation accuracy for varying sample sizes. ((II)) Imputation accuracy for varying sample sizes.

Fig B: Imputation accuracy of Scelestial.
(I) The number of missing values is shown as a blue dotted line and the number of incorrect imputations by Scelestial is shown in red. Thus the distance between the blue and the red line shows the number of correct imputations by Scelestial. (II) The incorrect imputation ratio of Scelestial. Note that error bars are too small to be seen in the first chart.

C Extended comparison of Scelestial with the other methods on OncoNEM simulated dataset

Table A represents performance comparison of the methods for larger sample size.

Table A: Extension of comparison of reconstruction of ground truth lineage tree from data simulated by OncoNEM, showing the distance between the inferred trees and the ground truth for all methods across eight lineage trees. The best results among all the methods for each evolutionary tree are shown in bold.

	150 samples				200 samples			
	5 clones		10 clones		5 clones		10 clones	
Method	20 sites	50 sites	20 sites	50 sites	20 sites	50 sites	20 sites	50 sites
OncoNEM	0.90	0.77	0.82	0.75	1.05	0.88	0.79	0.87
Scelestial	**0.81**	**0.72**	0.73	**0.68**	0.94	**0.77**	**0.73**	**0.78**
BitPhylogeny	0.87	0.76	0.88	0.96	0.94	0.90	0.92	1.13
SCITE	0.90	0.75	0.87	1.05	0.95	0.84	0.92	0.86
SASC	0.87	0.81	0.82	0.78	1.00	0.84	0.80	0.82
SCIPhI	0.84	0.88	0.97	0.88	1.00	0.86	0.88	0.87
SiFit	0.83	**0.72**	**0.71**	0.72	**0.92**	0.80	0.78	0.84
SiCloneFit	0.94	0.85	0.88	0.75	1.10	0.88	0.80	0.91

D Extended evaluation of Scelestial over a grid of parameters

We evaluated the tree reconstructions of Scelestial over many combinations of parameters for missing values, false positive and false negative rates, the number of samples and the number of sites (Table B (sample distance measure), Table C (split similarity measure)).

We evaluated the performance of Scelestial for changing all of the parameters over ranges of values. We used the following ranges: false positive rate: 0–8%; false negative rate: 0–40%; missing value rate: 0–40%; samples: 25–125; sites: 20–820. These values were chosen to range around the values we observed for single-cell datasets (Section 2.2 of the main manuscript). For each combination of parameters we evaluated the performance of Scelestial on one simulated dataset.

The results show that the sample distance measure increases to 72% and the split similarity measure decreases to 73% when the rate of missing values is 40%. In the common range of the values (false positive rate 0 to 3%, false negative rate 0 to 20%, missing value rate 0 to 20%, and 20 to 820 locus), however, the maximum sample distance measure is 53% and the minimum split similarity measure is 86%.

			locus	sample																	
			20				220				420				620			820			**Total Result**
Missing value rate	False negative rate	False positive rate	25	45	85	125	25	45	85	125	25	45	85	125	25	45	85	25	45	85	
0	0	0	0.39	0.12	0.39	0.52	0.12	0.05	0.06	0.06	0.07	0.06	0.15	0.05	0.08	0.11	0.14	0.10	0.08	0.09	**0.15**
		0.02	0.45	0.60	0.40	0.46	0.21	0.16	0.21	0.16	0.24	0.20	0.21	0.24	0.22	0.23	0.30	0.27	0.33	0.26	**0.28**
		0.04	0.52	0.56	0.57	0.47	0.24	0.26	0.26	0.28	0.27	0.34	0.27	0.28	0.35	0.24	0.27	0.31	0.30	0.34	**0.34**
		0.06	0.39	0.44	0.67	0.52	0.22	0.29	0.25	0.35	0.37	0.30	0.32	0.24	0.29	0.29	0.32	0.36	0.38	0.25	**0.35**
		0.08	0.56	0.39	0.47	0.50	0.24	0.18	0.23	0.25	0.27	0.28	0.27	0.31	0.33	0.49	0.42	0.45	0.39	0.41	**0.36**
	0.2	0	0.55	0.53	0.59	0.50	0.27	0.24	0.27	0.26	0.20	0.32	0.25	0.29	0.29	0.31	0.34	0.20	0.27	0.26	**0.33**
		0.02	0.36	0.53	0.42	0.44	0.30	0.23	0.32	0.22	0.27	0.33	0.34	0.35	0.26	0.29	0.29	0.41	0.36	0.30	**0.33**
		0.04	0.53	0.28	0.45	0.41	0.22	0.31	0.32	0.29	0.31	0.30	0.29	0.27	0.46	0.31	0.36	0.35	0.40	0.33	**0.34**
		0.06	0.51	0.57	0.46	0.44	0.37	0.31	0.29	0.46	0.39	0.51	0.40	0.44	0.38	0.33	0.38	0.43	0.36	0.53	**0.42**
		0.08	0.51	0.51	0.41	0.41	0.30	0.35	0.38	0.33	0.44	0.44	0.45	0.41	0.52	0.41	0.40	0.52	0.47	0.51	**0.43**
	0.4	0	0.56	0.54	0.51	0.60	0.44	0.47	0.47	0.32	0.41	0.40	0.36	0.36	0.32	0.45	0.45	0.50	0.36	0.41	**0.44**
		0.02	0.74	0.58	0.46	0.83	0.37	0.44	0.43	0.43	0.38	0.44	0.43	0.47	0.51	0.44	0.55	0.44	0.49	0.38	**0.49**
		0.04	0.67	0.61	0.52	0.52	0.50	0.29	0.45	0.46	0.35	0.46	0.41	0.53	0.41	0.48	0.45	0.37	0.46	0.51	**0.47**
		0.06	0.52	0.53	0.46	0.64	0.40	0.45	0.41	0.41	0.32	0.48	0.43	0.38	0.51	0.52	0.50	0.62	0.57	0.40	**0.47**
		0.08	0.52	0.57	0.70	0.54	0.53	0.54	0.56	0.56	0.57	0.49	0.47	0.51	0.48	0.58	0.54	0.48	0.45	0.49	**0.53**
0.2	0	0	0.45	0.54	0.39	0.51	0.39	0.30	0.27	0.31	0.36	0.31	0.39	0.28	0.37	0.25	0.31	0.34	0.40	0.35	**0.36**
		0.02	0.46	0.37	0.58	0.51	0.35	0.33	0.31	0.21	0.27	0.30	0.26	0.26	0.43	0.34	0.34	0.53	0.37	0.37	**0.37**
		0.04	0.55	0.45	0.53	0.21	0.33	0.27	0.33	0.33	0.28	0.32	0.28	0.32	0.36	0.37	0.30	0.35	0.42	0.41	**0.36**
		0.06	0.38	0.49	0.49	0.44	0.36	0.29	0.33	0.38	0.36	0.30	0.27	0.28	0.39	0.33	0.43	0.42	0.38	0.35	**0.37**
		0.08	0.39	0.47	0.48	0.50	0.37	0.36	0.27	0.25	0.36	0.27	0.41	0.32	0.44	0.38	0.33	0.52	0.35	0.32	**0.38**
	0.2	0	0.46	0.45	0.52	0.48	0.29	0.35	0.28	0.39	0.36	0.36	0.33	0.28	0.47	0.38	0.38	0.46	0.28	0.33	**0.38**
		0.02	0.59	0.41	0.55	0.44	0.50	0.32	0.37	0.30	0.30	0.31	0.31	0.30	0.45	0.34	0.35	0.37	0.46	0.51	**0.40**
		0.04	0.46	0.55	0.37	0.56	0.39	0.40	0.32	0.34	0.36	0.34	0.31	0.37	0.47	0.33	0.46	0.42	0.45	0.48	**0.41**
		0.06	0.66	0.56	0.55	0.35	0.38	0.43	0.37	0.32	0.46	0.33	0.45	0.36	0.42	0.39	0.49	0.45	0.49	0.42	**0.44**
		0.08	0.59	0.45	0.46	0.55	0.35	0.44	0.36	0.46	0.48	0.38	0.41	0.49	0.43	0.46	0.42	0.50	0.49	0.50	**0.46**
	0.4	0	0.63	0.50	0.52	0.45	0.44	0.60	0.47	0.41	0.48	0.43	0.54	0.41	0.56	0.60	0.56	0.40	0.45	0.49	**0.50**
		0.02	0.56	0.64	0.59	0.51	0.43	0.33	0.41	0.40	0.39	0.45	0.49	0.53	0.45	0.49	0.48	0.33	0.42	0.48	**0.47**
		0.04	0.57	0.49	0.62	0.63	0.51	0.45	0.39	0.41	0.48	0.51	0.45	0.49	0.46	0.47	0.55	0.47	0.52	0.50	**0.50**
		0.06	0.60	0.54	0.62	0.64	0.66	0.45	0.49	0.48	0.47	0.56	0.58	0.48	0.54	0.50	0.48	0.47	0.55	0.46	**0.53**
		0.08	0.55	0.56	0.59	0.55	0.51	0.42	0.44	0.54	0.54	0.53	0.50	0.53	0.52	0.53	0.55	0.60	0.61	0.56	**0.54**
0.4	0	0	0.55	0.63	0.55	0.56	0.35	0.51	0.33	0.29	0.48	0.30	0.51	0.39	0.38	0.39	0.41	0.42	0.42	0.37	**0.44**
		0.02	0.56	0.62	0.54	0.48	0.27	0.33	0.35	0.25	0.36	0.47	0.34	0.44	0.50	0.35	0.32	0.41	0.37	0.42	**0.41**
		0.04	0.47	0.64	0.40	0.50	0.33	0.32	0.36	0.32	0.37	0.33	0.51	0.50	0.45	0.36	0.50	0.40	0.38	0.50	**0.42**
		0.06	0.46	0.50	0.41	0.49	0.37	0.44	0.29	0.33	0.42	0.39	0.38	0.47	0.39	0.36	0.49	0.47	0.45	0.43	**0.42**
		0.08	0.52	0.58	0.50	0.59	0.35	0.33	0.41	0.27	0.53	0.59	0.43	0.43	0.56	0.38	0.46	0.50	0.59	0.57	**0.48**
	0.2	0	0.59	0.49	0.60	0.46	0.38	0.34	0.50	0.41	0.47	0.59	0.39	0.40	0.49	0.55	0.50	0.43	0.51	0.49	**0.48**
		0.02	0.65	0.58	0.53	0.53	0.36	0.42	0.48	0.39	0.58	0.38	0.48	0.43	0.49	0.50	0.48	0.39	0.39	0.54	**0.48**
		0.04	0.61	0.61	0.55	0.56	0.35	0.39	0.53	0.39	0.61	0.57	0.52	0.50	0.58	0.55	0.50	0.56	0.56	0.54	**0.53**
		0.06	0.57	0.52	0.66	0.48	0.53	0.38	0.48	0.44	0.52	0.52	0.49	0.54	0.52	0.60	0.51	0.62	0.49	0.50	**0.52**
		0.08	0.59	0.55	0.60	0.49	0.41	0.44	0.51	0.45	0.42	0.47	0.52	0.45	0.44	0.52	0.50	0.60	0.54	0.55	**0.50**
	0.4	0	0.50	0.57	0.56	0.60	0.50	0.51	0.56	0.60	0.57	0.50	0.59	0.54	0.60	0.53	0.57	0.56	0.54	0.63	**0.56**
		0.02	0.65	0.67	0.63	0.56	0.61	0.65	0.57	0.52	0.48	0.59	0.57	0.51	0.67	0.55	0.57	0.51	0.63	0.59	**0.58**
		0.04	0.67	0.57	0.61	0.67	0.61	0.53	0.61	0.57	0.58	0.55	0.57	0.58	0.65	0.64	0.58	0.54	0.56	0.60	**0.59**
		0.06	0.55	0.53	0.65	0.61	0.53	0.49	0.63	0.54	0.63	0.57	0.52	0.60	0.52	0.66	0.64	0.54	0.58	0.59	**0.58**
		0.08	0.58	0.55	0.64	0.72	0.51	0.69	0.54	0.59	0.61	0.60	0.58	0.59	0.50	0.54	0.65	0.65	0.54	0.62	**0.59**
**Total Result**			**0.54**	**0.52**	**0.53**	**0.52**	**0.39**	**0.38**	**0.39**	**0.37**	**0.41**	**0.41**	**0.41**	**0.40**	**0.44**	**0.42**	**0.44**	**0.45**	**0.44**	**0.44**	**0.44**

Table B: Robustness of Scelestial to variation in the properties of ground truth lineage trees in terms of sample distance in the trees between the inferred and ground truth trees.

Cells are colored with a color reflecting the value of the error, error value 1.00 is colored as 1.00 and the value 0.00 is colored as 0.00.

			locus	sample																
			20				220				420				620			820			**Total Result**
Missing value rate	False negative rate	False positive rate	25	45	85	125	25	45	85	125	25	45	85	125	25	45	85	25	45	85	
0	0	0	0.96	1.00	0.92	0.83	1.00	1.00	1.00	1.00	1.00	1.00	1.00	1.00	1.00	1.00	1.00	1.00	1.00	1.00	**0.98**
		0.02	0.84	0.88	0.96	0.93	1.00	1.00	1.00	1.00	1.00	1.00	1.00	1.00	1.00	1.00	1.00	1.00	1.00	1.00	**0.98**
		0.04	0.88	0.90	0.91	0.93	1.00	1.00	1.00	1.00	1.00	1.00	1.00	1.00	1.00	1.00	1.00	0.98	0.98	1.00	**0.98**
		0.06	0.93	0.92	0.86	0.90	1.00	1.00	1.00	1.00	0.99	1.00	1.00	1.00	0.99	1.00	1.00	0.96	0.97	1.00	**0.97**
		0.08	0.89	0.96	0.92	0.91	1.00	1.00	1.00	1.00	0.99	1.00	1.00	1.00	0.98	0.91	0.96	0.94	0.92	0.98	**0.96**
	0.2	0	0.82	0.90	0.89	0.94	1.00	1.00	1.00	1.00	1.00	1.00	1.00	1.00	1.00	1.00	1.00	1.00	1.00	1.00	**0.97**
		0.02	0.94	0.88	0.95	0.92	1.00	1.00	1.00	1.00	1.00	0.95	0.99	0.96	1.00	1.00	1.00	0.97	0.97	1.00	**0.97**
		0.04	0.87	0.99	0.95	0.94	1.00	1.00	1.00	1.00	0.98	1.00	0.99	1.00	0.94	0.96	0.97	0.97	0.95	0.98	**0.97**
		0.06	0.87	0.88	0.91	0.94	0.98	0.98	0.97	0.97	0.98	0.92	0.98	0.93	0.94	0.97	0.97	0.94	0.96	0.86	**0.94**
		0.08	0.90	0.90	0.93	0.93	0.98	0.98	0.96	0.99	0.95	0.96	0.94	0.93	0.92	0.93	0.94	0.84	0.90	0.88	**0.93**
	0.4	0	0.91	0.88	0.89	0.86	0.94	1.00	0.93	0.96	0.89	0.98	1.00	0.99	0.98	0.91	0.97	0.89	0.97	0.99	**0.94**
		0.02	0.82	0.87	0.95	0.79	0.98	0.91	0.96	0.92	0.99	0.91	0.94	0.92	0.90	0.92	0.89	0.91	0.93	0.93	**0.91**
		0.04	0.81	0.84	0.90	0.87	0.90	0.99	0.94	0.93	0.98	0.85	0.91	0.91	0.91	0.90	0.92	0.92	0.86	0.92	**0.90**
		0.06	0.91	0.87	0.89	0.86	0.93	0.94	0.95	0.92	0.97	0.91	0.94	0.95	0.89	0.82	0.86	0.84	0.80	0.90	**0.90**
		0.08	0.89	0.86	0.84	0.89	0.92	0.89	0.87	0.85	0.79	0.91	0.89	0.86	0.89	0.89	0.83	0.86	0.92	0.85	**0.87**
0.2	0	0	0.91	0.88	0.96	0.87	0.98	1.00	1.00	1.00	0.98	1.00	1.00	1.00	1.00	1.00	0.97	0.97	0.96	0.98	**0.97**
		0.02	0.91	0.98	0.90	0.89	1.00	1.00	1.00	1.00	1.00	1.00	0.99	1.00	0.97	1.00	1.00	0.91	0.97	0.99	**0.97**
		0.04	0.85	0.96	0.92	1.00	1.00	1.00	1.00	1.00	0.99	1.00	1.00	1.00	1.00	0.98	1.00	0.98	0.98	0.94	**0.98**
		0.06	0.96	0.92	0.94	0.96	0.98	1.00	1.00	0.99	0.94	1.00	1.00	1.00	0.98	0.98	0.96	0.92	0.98	0.97	**0.97**
		0.08	0.98	0.94	0.95	0.90	1.00	1.00	1.00	1.00	0.98	0.99	0.94	1.00	0.92	0.98	0.99	0.90	0.97	0.98	**0.97**
	0.2	0	0.93	0.90	0.92	0.93	0.99	0.97	1.00	0.96	0.98	0.96	1.00	1.00	0.93	0.96	0.98	0.94	0.99	0.97	**0.96**
		0.02	0.86	0.93	0.88	0.91	0.94	1.00	1.00	0.99	1.00	1.00	0.98	0.97	0.97	0.99	0.98	0.94	0.88	0.93	**0.95**
		0.04	0.94	0.92	0.98	0.90	0.98	0.99	0.99	0.98	0.97	0.94	0.99	0.96	0.88	0.98	0.91	0.94	0.92	0.90	**0.95**
		0.06	0.89	0.93	0.91	0.96	0.95	0.96	0.99	0.97	0.94	0.97	0.92	0.97	0.94	0.96	0.88	0.94	0.96	0.93	**0.94**
		0.08	0.88	0.95	0.94	0.86	0.98	0.93	0.94	0.92	0.96	0.98	0.93	0.93	0.94	0.96	0.95	0.89	0.91	0.92	**0.93**
	0.4	0	0.83	0.90	0.91	0.94	0.94	0.95	0.92	0.94	0.93	0.94	0.88	0.91	0.83	0.85	0.83	0.96	0.90	0.85	**0.90**
		0.02	0.89	0.81	0.83	0.88	1.00	0.98	0.95	0.96	0.96	0.94	0.85	0.83	0.91	0.90	0.90	0.98	0.95	0.90	**0.91**
		0.04	0.87	0.88	0.80	0.86	0.90	0.93	0.94	0.94	0.88	0.90	0.92	0.88	0.94	0.93	0.87	0.94	0.84	0.88	**0.89**
		0.06	0.83	0.91	0.86	0.80	0.81	0.94	0.87	0.90	0.89	0.87	0.84	0.87	0.88	0.88	0.88	0.90	0.89	0.88	**0.87**
		0.08	0.84	0.87	0.83	0.88	0.91	0.94	0.90	0.87	0.87	0.87	0.87	0.86	0.87	0.88	0.82	0.83	0.81	0.80	**0.86**
0.4	0	0	0.90	0.89	0.89	0.88	0.98	0.94	0.98	1.00	0.96	0.98	0.93	0.96	0.94	0.96	0.94	0.91	0.94	0.95	**0.94**
		0.02	0.86	0.87	0.88	0.95	0.98	1.00	1.00	0.99	0.94	0.88	0.98	0.93	0.91	0.94	0.99	0.93	0.96	0.96	**0.94**
		0.04	0.90	0.84	0.96	0.92	0.99	0.98	0.99	0.99	0.96	0.99	0.91	0.96	0.93	0.96	0.88	0.97	0.95	0.91	**0.94**
		0.06	0.95	0.93	0.93	0.89	0.93	0.92	0.99	0.99	0.92	0.96	0.96	0.92	0.94	0.96	0.88	0.96	0.92	0.95	**0.94**
		0.08	0.89	0.88	0.92	0.85	0.98	0.94	0.95	0.99	0.83	0.85	0.95	0.94	0.86	0.96	0.94	0.89	0.82	0.91	**0.91**
	0.2	0	0.86	0.90	0.86	0.91	0.95	0.96	0.91	0.94	0.91	0.86	0.95	0.93	0.86	0.88	0.89	0.93	0.87	0.89	**0.90**
		0.02	0.81	0.89	0.88	0.91	0.96	0.92	0.94	0.96	0.86	0.92	0.91	0.93	0.87	0.89	0.92	0.90	0.94	0.87	**0.90**
		0.04	0.84	0.83	0.88	0.88	0.94	0.97	0.88	0.96	0.86	0.83	0.87	0.89	0.86	0.86	0.88	0.86	0.85	0.86	**0.88**
		0.06	0.82	0.91	0.89	0.91	0.87	0.92	0.92	0.92	0.89	0.90	0.89	0.86	0.87	0.88	0.88	0.86	0.90	0.88	**0.89**
		0.08	0.84	0.91	0.86	0.89	0.90	0.91	0.89	0.91	0.90	0.88	0.84	0.91	0.86	0.89	0.90	0.88	0.88	0.86	**0.88**
	0.4	0	0.86	0.82	0.86	0.87	0.89	0.86	0.84	0.79	0.81	0.86	0.84	0.84	0.84	0.84	0.82	0.84	0.86	0.78	**0.84**
		0.02	0.76	0.81	0.82	0.85	0.85	0.85	0.85	0.87	0.86	0.81	0.81	0.85	0.80	0.85	0.83	0.84	0.80	0.81	**0.83**
		0.04	0.80	0.84	0.84	0.76	0.83	0.87	0.84	0.84	0.81	0.82	0.82	0.81	0.73	0.81	0.83	0.84	0.82	0.83	**0.82**
		0.06	0.83	0.86	0.77	0.81	0.86	0.87	0.81	0.85	0.82	0.81	0.88	0.81	0.84	0.78	0.80	0.79	0.83	0.82	**0.83**
		0.08	0.83	0.85	0.82	0.76	0.90	0.81	0.82	0.80	0.79	0.84	0.82	0.82	0.83	0.84	0.80	0.76	0.86	0.83	**0.82**
**Total Result**			**0.87**	**0.89**	**0.89**	**0.89**	**0.95**	**0.96**	**0.95**	**0.95**	**0.93**	**0.93**	**0.93**	**0.93**	**0.92**	**0.93**	**0.92**	**0.91**	**0.92**	**0.91**	**0.92**

Table C: Robustness of Scelestial to variation in the properties of ground truth lineage trees in terms of topological similarity between the inferred and ground truth trees.

Cells are colored with a color reflecting the value of the similarity, similarity value 1.00 is colored as 1.00 and the value 0.50 is colored as 0.50. Although the values potentially could be in range between zero and one, however since there is no similarity value less than 0.50 in this table, we used range of colors for similarity values between 1.00 and 0.50.

E Identifying mutations in single-cell datasets of the case studies

The single-cell datasets used for the case study were obtained from [1,2]. In this section, we briefly summarize the methods of generating the datasets.

The data for the "Case study: a single-cell dataset from a muscle-invasive bladder tumor" (Section 2.3 of the main manuscript) were obtained from [1]. The tissue was digested by collagenase I and IV, and single cells were randomly selected from the tumor tissue and normal adjacent tissue. Exome capture was performed on the whole-genome amplification (WGA) products of each cell. Second-generation sequencing was then performed on the resulting libraries by Illumina HiSeq 2000 with the paired-end 100 bp read option. To reduce errors in the subsequent analyses, cells with a coverage of less than 70% of the exome targets or a significant false heterozygous rate across the X chromosome were discarded. Among 66 sequenced cells, 44 single cells from the tumor tissue (BC cells) and 11 from the normal adjacent tissue (BN cells) were selected. The average sequencing depth in the exome regions of the qualified single cells was 40-fold, which equals approximately 2,200-fold coverage from all cells. Reads were uniquely mapped to exome region of Human genome and 100 bp-flanking regions selected for SNP calling by the following criteria (1) a Q20 score quality cutoff; (2) at least five reads; (3) a p-value >0.01; (4) 5 bp distance between them; and (5) 1/3   3 variation between quality score of two bases in heterozygous sites. Genotype calling was successful for the majority of sites in the exome regions [3]. On average, 88.6% whole-exome coverage of all qualified single cells was achieved, and more than 60% of the target region was covered with greater than 5x sequencing depth in all cells. The whole exome of bulk DNA from the same bladder cancer tissue with a coverage of 137x and the normal bladder tissue with a coverage of 28x was sequenced, to be used for quality control of single-cell sequencing. A site that was identified as homozygous in all normal cells with a minimum coverage of six

reads but had mutants in less than three cancer cells was called a somatic mutation [4, 5]. The threshold used in this definition for the number of normal cells was calculated via a binomial test with the parameters of the obtained allelic dropout value, the number of qualified normal cells, and the whole-exome size. The threshold for the number of cancer cells was calculated via a binomial test adapted for the false discovery rate, the number of qualified cancer cells, and the whole-exome size. In total, from the single-cell exomes and their 100 bp flanking regions, 443 somatic mutations were identified.

The data for the "Case study 2: a single-cell dataset from metastatic colorectal cancer" (Section 2.4 of the main manuscript) were obtained from [2] through single-cell multiple-displacement-amplification (MDA) with a 2:3 ratio of lysis buffer (200 mM KOH, 50 mM DTT):1× PBS solution. Single-cell libraries were barcoded for highly multiplexed targeted sequencing with a 1000 cancer gene panel (T1000) that captures 12,500 exons and promoter regions [6]. To distinguish germlines from somatic mutations, matched normal tissue was sequenced. The exome libraries were sequenced at a high coverage depth (75.5x) and breadth (97.33%). The breadth rate is defined as the ratio of the targeted region with a physical coverage of at least 1x depth. GATK [7] was used for variant calling, and sites with less than 10x coverage were reported as missing values. A site was considered as a variant if the number of detected variants was higher than a threshold, based on the depth of its coverage. The threshold used for sites with coverage 10–20x, 20–100x, and 100–250x, was 10 variant reads, 30% of variant reads, and 20% of variant reads, respectively.In total, 127 mutations were identified in Patient 1, and 131 mutations were identified in Patient 2.

F Details of comparison of methods on real datasets

Phylogenies generated by different methods are described in the manuscript (Figs 1, 5, and 6 of the main manuscript) and their properties are discussed there (Sections 2.3 and 2.4 of the main manuscript). In this section a formal definition of the measures and corresponding values for different methods and datasets is given (Table 2).

- Mixed cancer and normal sample: Number of samples mixed in a colony containing both normal and cancer samples.
- Normal sample with cancer sample parent: For each sample, consider the nearest parent to which a sample is assigned. We name the parent as the first parent sample. For this measure, we calculated the number of normal or mixed nodes with a cancer sample or a mixed cancer and normal sample colony as the first parent sample. Note that a colony is counted at most once for this measure.
- Cancer sample components: Removing all the colonies with only normal samples, their direct parents, and all the edges with one end connected to mixed colonies results in a directed graph. This graph, after removing components only containing internal nodes without assigned samples, is called the cancer component graph. The number of components of this graph is the cancer sample component measure.
- Removing all normal samples and their direct parents if no sample is assigned to them leads to a set of cancer sample components. The "cancer sample components" measure calculates the number of these components. Note that we keep mixed colonies but remove their parents if no sample is assigned to them.
- Cancer samples not in the largest component: Number of samples not in the largest component of cancer samples. Cancer sample components are defined in the "Cancer sample components" measure.

Overall, Scelestial and SiCloneFit showed the best performance for the dataset of muscle-invasive bladder tumor, with better or equal performance to other methods in all the measures, and OncoNEM stands next to them (Table 2). For the first patient of metastatic colorectal cancer dataset, SiCloneFit shows the best performance and Scelestial has the second-best performance. For patient 2 of the same dataset, Scelestial shows the best and SiCloneFit the second-best performance.

G Extended performance and runtime analysis of the methods on large datasets

Simulated and real datasets analyzed in our and related work [8–10] have sample sizes of less than 500 and fewer than 100 mutations. However, new technologies allow hundreds of samples and hundreds of mutations. In this section, we compare the methods over large datasets (Figs C, D, and E).

On large datasets, both in number of sites and number of samples, Scelestial and SiCloneFit have best performance with respect to split similarity measure and lineage tree reconstruction error (Figs C and D). Similar to the same observation for normal-size samples (Fig 8 of the main manuscript) Scelestial's results have smaller variance in comparison to SiCloneFit. Note that OncoNEM existed with errors on datasets with high numbers of samples.

From the running time point of view, SASC, SiCloneFit, SCITE have higher running time in comparison to SCIPhI, BitPhylogeny, and Scelestial (Fig E(II)). Among these three fast methods, Scelestial shows best performance both in split similarity and lineage tree reconstruction error measures (Fig C). On datasets with high numbers of samples, the two methods with highest accuracy, Scelestial and SiCloneFit, in addition to SASC are the slowest ones. In this range of number of samples (500-900), run time of Scelestial highly increases and at 800 samples Scelestial became slower than SiCloneFit (Fig E(I)). Accuracy of the methods are similar to their accuracy on a large number of sites (Fig D), i. e. Scelestial and SiCloneFit show best performance with respect to lineage tree reconstruction error and sample distance measures. In conclusion, considering accuracy and run time, Scelestial and SiCloneFit are the best options. While two methods show similar accuracy, on datasets with high number of sites Scelestial is faster and on datasets with high number of samples (800 samples or more) SiCloneFit is a faster option. Note that in comparison to SiCloneFit, Scelestial shows less variation in the presented measures on different datasets.

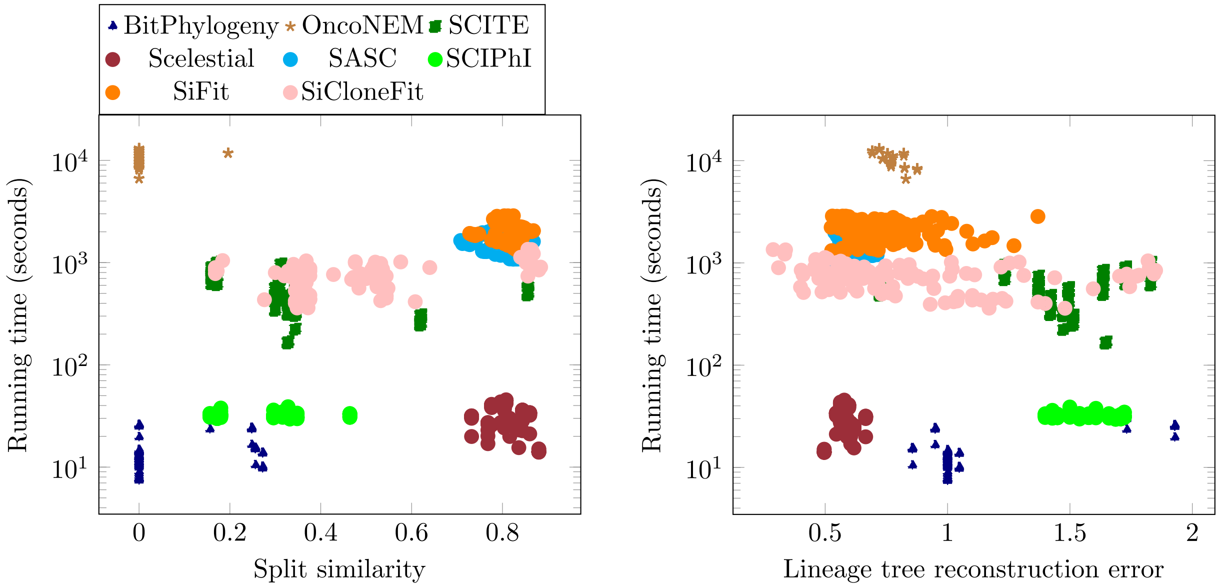

((I)) Run time and lineage reconstruction error of four lineage tree inference methods on datasets with different numbers of sites with respect to the pair distance error.

((II)) Run times and lineage reconstruction errors of four methods on datasets with different numbers of sites with respect to the split similarity measure.

Fig C: Comparison of methods with respect to running time and lineage tree reconstruction error on simulated datasets of varying number of sites. The dataset is generated with the simulation method provided in Section 3.2 of the main manuscript. The data contains 50 samples, 1000 to 2000 sites (with step size of 100), missing value rate 7%, false positive rate 1.5%, and false negative rate 10%.
Note that on this dataset, OncoNEM exited with error on all the tests.

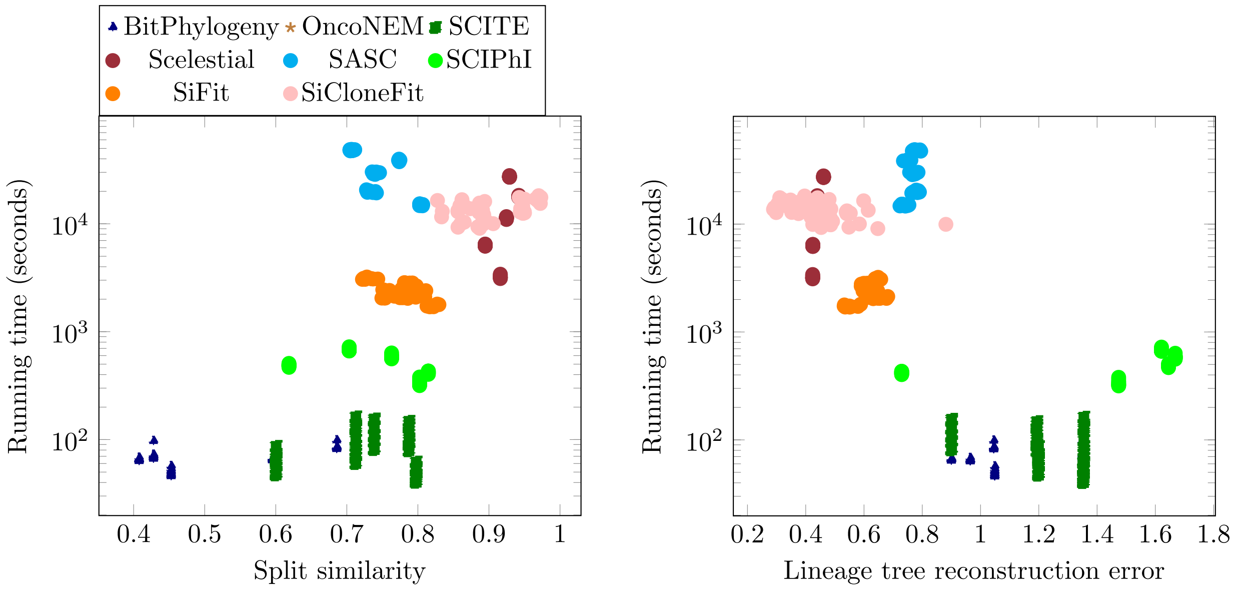

((I)) Run time and lineage reconstruction error of four lineage tree inference methods on datasets with different numbers of sites with respect to the pair distance error.

((II)) Run times and lineage reconstruction errors of four methods on datasets with different numbers of sites with respect to the split similarity measure.

Fig D: Comparison of methods with respect to running time and lineage tree reconstruction error on simulated datasets of varying sample size. The dataset is generated with the simulation method provided in Section 3.2 of the main manuscript. The data contains 500 to 900 samples (with size of 100), 100 sites, missing value rate 7%, false positive rate 1.5%, and false negative rate 10%.

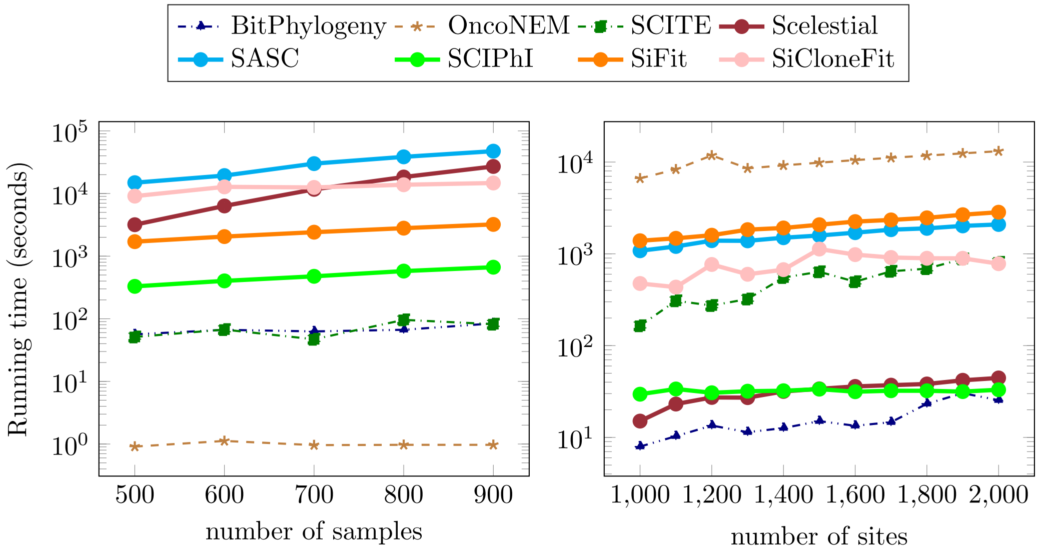

((I)) Run time comparisons for varying sample sizes.

((II)) Relation of run time to the number of sites

Fig E: Run time comparison in relation to the number of samples and sites. (a) Simulated data presented in Fig D, (b) Simulated data presented in Fig C.

H Comparing the performance evaluation of the methods in this study to previous studies

SCITE, SiFit, SiCloneFit, and OncoNEM were evaluated on simulated datasets [8–10], including datasets consisting of single-cell sequencing data as well as bulk datasets, and simulated single-cell-only datasets.

The data simulated in [10] were generated under the k-Dollo model with k=1 with a low false positive rate and false negative rate. Although the data generated under this model are compatible with the assumptions under which the SPhyR was developed, for the measures (a) ancestral pair recall, (b) incomparable pair recall, and (c) clustered pair recall, the methods showed a performance below 65% or, in other words, showed more than 35% error. The percentage of 35% is equivalent to 0.7 of the sample distance error measure, which is a measure in range 0 to 2, and it is similar to the performance we obtained for the several methods in our study (Fig 8 of the main manuscript). The difference in performance comparison might be due to the k-Dollo assumption of the simulation in [10]. Note that SPhyR was not evaluated for non k-Dollo models.

The simulation provided in [8] was generated with a generative model over which the SiCloneFit's model is designed. Although the results in [8] demonstrate the excellent performance of all methods (SiCloneFit, SCG, OncoNEM, SiFit, and SCITE) in terms of the cluster accuracy and genotype reconstruction measures (Fig 2 A and 2B in [8]), the topological error of all the models except for SiCloneFit was larger than 1.

To correctly interpret the error measure evaluated in [8] , it is worth knowing that the error was calculated from the number (and not the length) of the edges between all the pairs of samples in the clonal tree, which has nine edges with non-zero lengths. The measures we used in our comparisons are more stringent than this measure. The result of performance analysis in [8] shows an average distance of 0.5 between pairs of samples on the

SiCloneFit's inferred trees, which each have nine edges. In a tree with nine edges, the distance between pairs of nodes is in the range of 2 to 9, and on a semi-balanced binary tree, this average distance is 2.54. Thus the average value of $|d_{ij}(I) - d_{ij}(T)|$ over random trees is 1.71. In comparison to the error value of 1.71 for a random tree, the average value of 0.7 obtained in [8] for SiCloneFit and ¿1.5 for OncoNEM, SiFit, and SCITE for 50 samples are relatively high and are compatible with the performance evaluation results shown in our manuscript. This calculation shows that the topological similarity measure for the methods OncoNEM, SiFit, and SCITE reported in [8] are not more than 1.14 times less than the average similarity between random trees. For the 100 sample assessment in that study, the algorithms performed better with respect to this measure, but the value of 0.5 for SiCloneFit and values ¿1 for OncoNEM, SiFit, and SCITE are still rather high. For the 100 samples, OncoNEM, SiFit, and SCITE were only 2times better than the average error for a random tree. However, the error for SiCloneFit was less, possibly due to the fact that simulated data was generated under compatible assumptions for SiCloneFit, though. Also in our evaluation SiCloneFit performed well (Fig 8 of the main manuscript).

The measures used in [9] are not similar to the measures we applied in our study, which focus on comparing the topology and branch lengths of two phylogenies, while their metrics "Pairwise ancestral relationship error" counts the number of misplaced mutations, and is only suitable for 1-Dollo models. Specifically, in [9], the measure "Pairwise ancestral relationship error" is used for a comparison of the methods. Although this measure is not independent of the topology of the trees, it fits best for the 1-Dollo model evaluation, and that is likely why SiFit was not included in Fig 2-middle chart in [9]. The other measure defined in the article, "Mutation matrix error", evaluates the number of mis-inferred mutations for each sample based on the 1-Dollo model and it does not consider the topology of the phylogenies directly.

The measures used in [9] are not similar to the measures we applied in

our study, which focus on comparing the topology and branch lengths of two phylogenies, while their metrics “Pairwise ancestral relationship error” counts the number of misplaced mutations, and is only suitable for 1-Dollo models. Specifically, in [9], the measure “Pairwise ancestral relationship error” is used for a comparison of the methods. Although this measure is not independent of the topology of the trees, it fits best for the 1-Dollo model evaluation, and that is likely why SiFit was not included in Fig 2-middle chart in [9]. The other measure defined in the article, “Mutation matrix error”, evaluates the number of mis-inferred mutations for each sample based on the 1-Dollo model and it does not consider the topology of the phylogenies directly.

I Extended analysis of Scelestial's phylogenetic tree for case studies

We analyzed the mutation matrices of the colorectal cancer single-cell data to characterize the misplacement of tumor cells in the tree inferred by Scelestial (Sections 2.2 and 2.3 of the main manuscript) for these datasets.

In the phylogeny inferred by Scelestial for the first patient (Fig 5d of the main manuscript) in the colorectal cancer single-cell dataset, the metastasis samples M-154 and M-155 were misplaced closer to normal samples in comparison with primary or metastasis samples. Although these two samples contained mutations in *TP53*, *APC*, and *KRAS*, which were common in cancer cells, these two samples have a mutation in *GATA1*, which was common in metastasis cells, sample M-154 have a mutation in *RBFOX1*, and sample M-155 had a mutation in *ZNF521*, which are common in metastasis cells. However, these two samples do not have mutations in *MYH9*, *CCNE1*, and *TCF7L2*, which are common to most other cancer cells. Mutations in *EYS*, *POU2AF1*, *ROBO2*, *TDRP*, and *FAT3* are mutations that are also common in other cancer cells but not in one of these two cells, and are missing values in the other one. These are the signals guiding Scelestial to misplace metastasis cells M-154 and M-155. In the same data, metastasis sample M-153 is misplaced closer to normal cells instead of the other metastatic cells. The mutation pattern of the cell M-153 is between that of primary tumor cells and normal cells. Mutations in *ZNF521*, *RBFOX1*, *TRRAP*, *GATA1*, *EYS*, and *TPM4* are common in metastasis cells from which only one (mutation in *GATA1*) is present in metastasis cancer sample M-153. Mutations in *MYH9*, *CCNE1*, *POU2AF1*, *ROBO2*, *TP53*, *APC*, *TDRP*, *FAT3*, *KRAS*, and *TCF7L2* are also common in the primary tumor cells of metastasis cell sample M-153 contains only four mutations (in *CCNE1*, *POU2AF1*, *TP53*, and *KRAS*). This pattern of mutations explains why Scelestial placed the metastasis cell samples M-153, M-154, and M-155 closer to normal cell than cancer

cell samples. A potential evolutionary scenario leading to this observation is that samples M-154 and M-155, independent of the other cancer cells, gained mutations *ZNF521*, *RBFOX1*, and *GATA1*.

In the phylogeny inferred by Scelestial (Fig 6b of the main manuscript) for the second patient in the colorectal cancer single-cell dataset, metastasis cell sample MP1-176 was misplaced closer to normal cells than to cancer cells. In this dataset, mutations 1–16 (in APC, FHIT, ATP7B, LINGO2, LRP1B, CHN1, IL21R, APC, TOX, MN1, MYH11, TP53, NRAS, CDK4, STRN) are common in primary tumor cells, and metastasis cell samples contain at least two mutations from mutations 17–30 (in LINGO2, IL7R, SPEN, F8, LAMB4, PIK3CG, PTPRD, FUS, NR4A3, HELZ, PRKCB, TSHZ3). Note that of the mutations 1–16, there were two mutations in *LINGO2* and of the mutations 17–30 there were three different mutations in *LINGO2*. Sample MP1-176 had three of the mutations 1–16 (in *NRAS*, *CDK4*, and *STRN*) and two missing values in this range of mutations. A potential evolutionary scenario for this phylogeny is the gain of at least three mutations of mutations 1–16 independent of the rest of the tumor.

J Scelestial's inferred phylogenies on case studies in a format with samples as leaf nodes

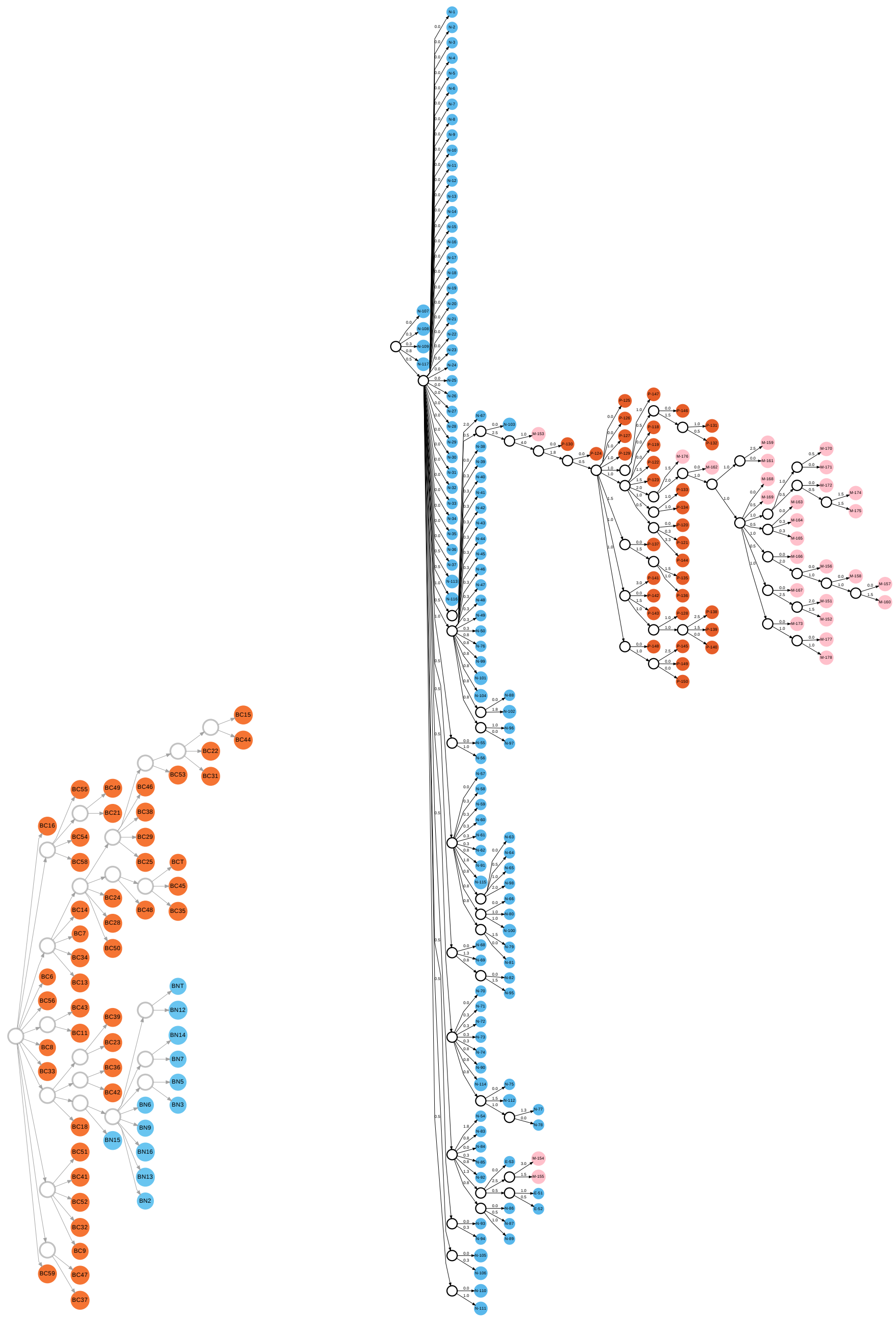

((I)) Scelestial's tree for single-cell dataset from a muscle-invasive bladder tumour

((II)) Scelestial's tree for single-cell dataset of the first colorectal cancer patient

Fig F: Scelestial phylogenies inferred for single-cell case studies corresponding to Fig 1, 5, and 6 of the main manuscript
This figure contains the tree generated by Scelestial with the "-no-internal-sample" flag. With this option samples are moved to leaf nodes and to internal nodes no sample is assigned. The general structure of the topology of the phylogeny does not change since the new leaf nodes are added with zero-length branch lengths to the tree."

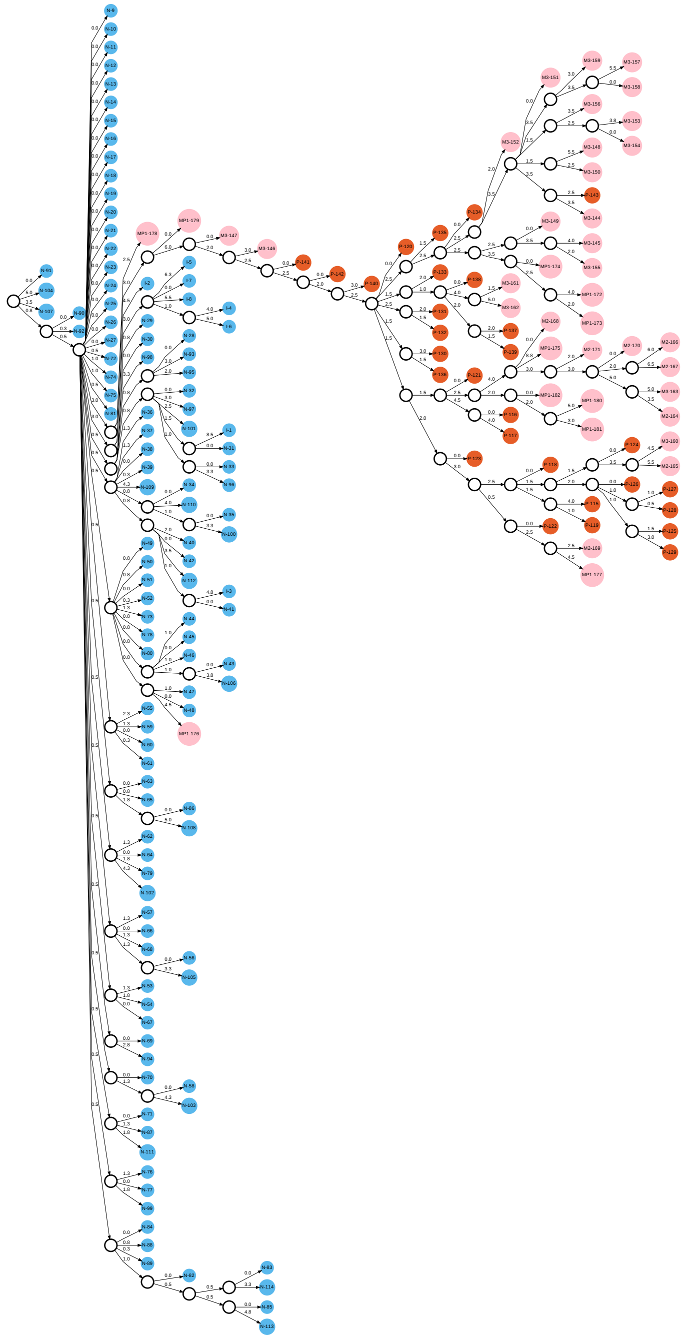

((III)) Scelestial's tree for single-cell dataset of the second colorectal cancer patient

Fig F: Scelestial phylogenies inferred for single-cell case studies corresponding to Figs 1, 5, and 6 of the main manuscript

References

1. Li Y, Xu X, Song L, Hou Y, Li Z, Tsang S, et al. Single-cell sequencing analysis characterizes common and cell-lineage-specific mutations in a muscle-invasive bladder cancer. GigaScience. 2012;1(1):12.

2. Leung ML, Davis A, Gao R, Casasent A, Wang Y, Sei E, et al. Single-cell DNA sequencing reveals a late-dissemination model in metastatic colorectal cancer. Genome Research. 2017;27(8):1287–1299.

3. Yi X, Liang Y, Huerta-Sanchez E, Jin X, Cuo ZXP, Pool JE, et al. Sequencing of 50 human exomes reveals adaptation to high altitude. science. 2010;329(5987):75–78.

4. Xu X, Hou Y, Yin X, Bao L, Tang A, Song L, et al. Single-cell exome sequencing reveals single-nucleotide mutation characteristics of a kidney tumor. Cell. 2012;148(5):886–895.

5. Hou Y, Song L, Zhu P, Zhang B, Tao Y, Xu X, et al. Single-cell exome sequencing and monoclonal evolution of a JAK2-negative myeloproliferative neoplasm. Cell. 2012;148(5):873–885.

6. Leung ML, Wang Y, Kim C, Gao R, Jiang J, Sei E, et al. Highly multiplexed targeted DNA sequencing from single nuclei. Nature protocols. 2016;11(2):214–235.

7. Van der Auwera GA, O'Connor BD. Genomics in the cloud: using Docker, GATK, and WDL in Terra. O'Reilly Media; 2020.

8. Zafar H, Navin N, Chen K, Nakhleh L. SiCloneFit: Bayesian inference of population structure, genotype, and phylogeny of tumor clones from single-cell genome sequencing data. Genome Research. 2019;29(11):1847–1859.

9. Satas G, Zaccaria S, Mon G, Raphael BJ. Scarlet: Single-cell tumor phylogeny inference with copy-number constrained mutation losses. Cell systems. 2020;10(4):323–332.

10. El-Kebir M. SPhyR: tumor phylogeny estimation from single-cell sequencing data under loss and error. Bioinformatics. 2018;34(17):i671–i679.
